# Quantitative Signal Characteristics of Electrocorticography and Stereoelectroencephalography: The Effect of Contact Depth

**DOI:** 10.1097/WNP.0000000000000577

**Published:** 2019-03-26

**Authors:** James J. Young, Joshua S. Friedman, Fedor Panov, Divaldo Camara, Ji Yeoun Yoo, Madeline C. Fields, Lara V. Marcuse, Nathalie Jette, Saadi Ghatan

**Affiliations:** Departments of * Neurology and; † Neurosurgery, Icahn School of Medicine at Mount Sinai, One Gustave L. Levy Place, New York, New York, U.S.A.

**Keywords:** Electrocorticography, Stereoelectroencephalogaphy, Spectral power, Phase–amplitude coupling, Epilepsy surgery

## Abstract

**Purpose:**

Patients undergoing epilepsy surgery often require invasive EEG, but few studies have examined the signal characteristics of contacts on the surface of the brain (electrocorticography, ECOG) versus depth contacts, used in stereoelectroencephalography (SEEG). As SEEG and ECOG have significant differences in complication rates, it is important to determine whether both modalities produce similar signals for analysis, to ultimately guide management of medically intractable epilepsy.

**Methods:**

Twenty-seven patients who underwent SEEG (19), ECOG (6), or both (2) were analyzed for quantitative measures of activity including spectral power and phase–amplitude coupling during approximately 1 hour of wakefulness. The position of the contacts was calculated by coregistering the postoperative computed tomography with a reconstructed preoperative MRI. Using two types of referencing schemes—local versus common average reference—the brain regions where any quantitative measure differed systematically with contact depth were established.

**Results:**

Using even the most permissive statistical criterion, few quantitative measures were significantly correlated with contact depth in either ECOG or SEEG contacts. The factors that predicted changes in spectral power and phase–amplitude coupling with contact depth were failing to baseline correct spectral power measures, use of a local rather than common average reference, using baseline correction for phase–amplitude coupling measures, and proximity of other grey matter structures near the region where the contact was located.

**Conclusions:**

The signals recorded by ECOG and SEEG have very similar spectral power and phase–amplitude coupling, suggesting that both modalities are comparable from an electrodiagnostic standpoint in delineation of the epileptogenic network.

## Abstract 2

Supplemental Digital Content is Available in the Text.

Although the surgical management of epilepsy without invasive monitoring can be considered in select cases,^1–5^ almost half of all surgical candidates require invasive EEG for the successful delineation of their seizure onset zone or epileptogenic network to establish the optimal therapeutic approach.^6–9^ At present, two modalities exist for performing invasive monitoring: electrocorticography (ECOG) or stereoelectroencephalography (SEEG).^10^ In ECOG, manually placed depth contacts may also be included but most contacts are placed on the cortical surface; SEEG uses stereotactically placed depth contacts and can sample bilaterally and disparate cortical regions inaccessible or practically unfeasible through subdural grid and strip electrode monitoring. There are significant differences in implementation and complication rate between the two procedures. For example, the infection risk for ECOG is 2.3% while the hemorrhage risk is 4%^1^ compared with 0.8% infection risk and 1% to 0.4% hemorrhage risk for SEEG.^2,11^ Direct electrical stimulation for mapping of eloquent cortex is usually performed using ECOG; however, there is some evidence to suggest that language mapping with SEEG gives concordant results to ECOG.^12^

Despite extensive experience with both techniques in the setting of epilepsy surgery, limited data exist to examine the characteristics of the signals observed by both modalities, particularly as they relate to the background activity. For example, interictal discharges appear to be sampled equally well by subdural and depth contacts.^13^ Depth recording in the hippocampus may offer superior lateralization to subdural contacts alone in the detection of seizures,^14^ but other series have showed best performance with combinations of contact types.^15^ The underlying assumption is that a SEEG contact placed close to a cortical surface is recording from that brain region, but no evidence exists about how close to a cortical surface the contact must be. In part, a dearth of information exists about what constitutes a “normal” background in the setting of intracranial EEG. Particular patterns of fast and rhythmic activity are associated with subtypes of focal cortical dysplasia^16^ or may help differentiate temporal from extemporal onsets.^17^ However, normal value ranges of spectral power for particular brain regions have only recently been compiled,^18^ and, although this study evaluated over 100 subjects, relatively brief duration recordings were used (60 seconds), and the effect of contact depth was not analyzed.

Further, as limited data are available that have directly compared the signals observed between ECOG and SEEG, the decision over which modality to use is highly variable between centers and is based on the epilepsy team's experience and the perception of benefit for each procedure for a specific patient. For example, ECOG is frequently noted to be superior to SEEG for lesions near eloquent cortex,^10^ but neuromodulatory rather than destructive options are increasingly being used by our center and others.^19,20^ While insular epilepsy can be interrogated using subdural strips,^21^ the use of ECOG in this setting requires an intrasylvian dissection; hence, SEEG may be preferable. Stereoelectroencephalography may obviate the need for a more invasive operation involving subdural recordings and provide greater utility by sampling from bilateral and distant cortical regions. In addition, from a research perspective, there is a broad neuroscience literature using data from epilepsy patients to understand the role of oscillatory activity in supporting cognition.^22–25^ However, different studies use ECOG or SEEG, and some studies report combinations of depth and subdural electrodes, but it is not clear whether a depth electrode located in white matter near a brain region provides a signal similar to a subdural strip. Hence, from a clinical perspective to understand the relative merits of the two modalities and from a scientific perspective to make studies with ECOG and SEEG mutually interpretable, a comparison of the signals between ECOG and SEEG is desirable.

To determine whether the intracranial EEG modality affects the quantitative characteristics of the recorded signal, we examined the intracranial EEGs from patients with SEEG, ECOG, or both. We computed spectral power and phase–amplitude coupling (PAC)—a measure of nested oscillations—for each contact. These measures were compared with the location of contacts to determine which measures, if any, correlated with contact depth. We hypothesized that SEEG contacts that were close to the cortical surface would record signals with very similar spectral power and PAC to ECOG contacts.

## METHODS

### Consent and Subjects

Consent was obtained from 26 consecutive adults (6 ECOG studies, 18 SEEG studies, and 2 who had both studies) undergoing invasive monitoring for the surgical management of drug-resistant epilepsy. The study was conducted according to the principles of the Declaration of Helsinki, and the consent documentation and procedure were approved by the Mount Sinai Hospital Institutional Review Board.

### Contact Localization

Localization of contacts was determined using preoperative volumetric MRIs and confirmed using postoperative volumetric computed tomographys. Coregistration of MRI and computed tomography was performed using iELVIS,^26^ and the location of each contact was selected on the postoperative computed tomography. A parcellated image of the patient's cortical surface was generated using FreeSurfer from the T1 series of the preoperative volumetric MRI.^27,28^ Cortical parcellation is by the DKT40 Atlas.^29^ Grid and strip contacts were projected onto the cortical surface using previously described methods.^30^ Depth contacts placed in the setting of an ECOG study were treated identically to the SEEG contacts. Stereoelectroencephalography contacts that were outside the brain parenchyma were excluded from this analysis.

To maximally distinguish any differences in activity that may exist with contact depth, the depth of each contact was calculated in four ways. First, the distance from the nearest gray matter structure was determined by starting at the voxel where the contact was located. Then a spiral search pattern was used to find the closest gray matter voxel. The distance between the centers of these two voxels was counted as the contact depth. This method is subsequently referred to as the voxel-wise depth. Second, the distance to the calculated pial and white matter surface are referred to as the pial depth and white matter depth, respectively. Finally, a grey matter proximity index was calculated for each contact. Grey matter proximity index is the distance between the contact and the nearest white matter surface divided by the cortical thickness in that point.^31^ For grid and strip contacts in ECOG studies, the value of each of these depths was treated as 0. Because brain regions such as the amygdala and hippocampus do not have pial contact, and hence do not have a pial depth, we excluded these brain regions from this analysis.

### Data Acquisition

Electrophysiological data were collected for all subjects using a Natus XLTEK128 or Natus Quantum amplifier (Natus Medical Incorporated, Pleasanton, CA). The sampling rates were between 512 and 2,048 Hz. Approximately 1 hour of wakefulness was pruned and confirmed by video monitoring of the subject. Any portion of the study that was within 12 hours of a seizure was excluded. Contacts that had interictal discharges, were within the patient's seizure onset zone, or were poorly connected by visual inspection were excluded from the analysis. All analysis was performed with MATLAB (Mathworks, Natick, MA) using the FieldTrip software library.^32^ Each trace was locally detrended using a first-order polynomial. Alternating current noise was removed using a 60-Hz notch filter. Again, to maximally distinguish any differences in activity that may exist with contact depth, referencing was performed in two ways. Either the contacts were re-referenced to the average of all contacts (common average reference, CAR), or the contacts were re-referenced to the average of their two neighbors (if the contact was a strip or a depth) or four neighbors (if the contact was a grid).^33^ Referencing to neighbors is subsequently referred to as local referencing (LR).

### Analysis

Quantitative measures including spectral power and PAC were selected because they are prevalent in the literature and are thought to encode information necessary to support cognition.^22,34,35^ Spectral power and PAC were also selected because unlike pairwise measures of activity, such as coherence, activity can be attributed to a single location, making direct comparison of the two modalities easier to interpret. The data from each contact were divided into 10-second windows with 5-second step-size. Spectral power was calculated using a complex Morlet wavelet transformation (width = 7 for 1–10 Hz, width = 14 for 11–30 Hz, and width = 21 for 31–150 Hz). Spectral power was calculated for all contacts between 1 and 150 Hz, calculated at 1 Hz increments. Phase–amplitude coupling—a measure of the tendency of high-frequency oscillations to occur at particular phases of low-frequency oscillations—was calculated for 1 to 20 Hz in 1 Hz increments (phase frequency) and 30 to 150 Hz in 10 Hz increments (amplitude frequency).^36^

### Statistics

For each brain region, data were analyzed only if there was at least one grid or strip contact available. The Pearson correlation (R) was calculated between the spectral power and PAC values and the contact depth using all four measures of contact depth. The analysis was repeated twice using both the CAR and the LR referencing schemes. Further, as prior comparisons of ECOG and SEEG recordings have shown that SEEG has systematically lower overall spectral power,^18^ the analysis was again repeated after baseline correction by Z-scoring the results of spectral power and PAC. This removes the effect of overall differences in signal amplitude and instead determines whether the shape of the spectrogram or PAC differs as a function of depth. The function of these repeated analyses—four measures of contact depth, two referencing schemes, with and without baseline correction using Z-scoring—is to maximize the possibility of detecting differences in activity with contact depth.

## RESULTS

### Characteristics of the Dataset

**FIG. 1.**
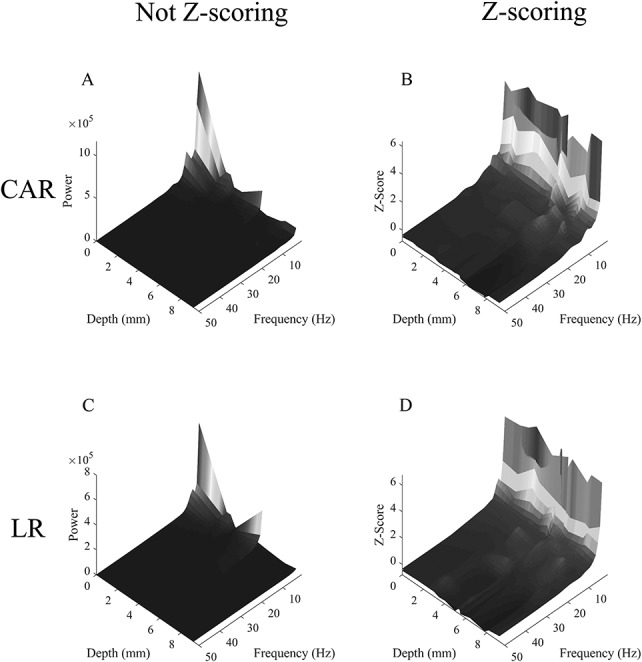
The spectral power (**A** and **C**) or baseline corrected spectral power by Z-scoring (**B** and **D**) in the left precentral gyrus for all included contacts as a function of depth and frequency. Warmer colors indicate a higher spectral power or Z-scored spectral power: (A) and (**B**) use CAR; (**C**) and (**D**) use LR. Note that the attenuation of frequencies that occurs with increasing depth is lessened in the Z-scored analyses (**B** and **D**). CAR, common average reference; LR, local referencing.

Twenty-one SEEG and eight ECOG approximately 1-hour studies were compared for quantitative characteristics of the signal. The patient demographics and clinical characteristics are listed in Table 1. There was a total of 825 contacts without interictal discharges outside the seizure onset zone. Using CAR, 634 contacts were included in the analysis—including 498 SEEG, 8 depth contacts, 97 grid, and 31 strip contacts. Using LR, 517 contacts were used for analysis. There are fewer contacts using LR because contacts were excluded if they did not have a full local montage. The subject number, location, voxel-wise depth, and type of contact are listed in **Supplemental Digital Content 1** (see **Table 1**, http://links.lww.com/JCNP/A57). An example showing the spectral power at different depths for the left precentral gyrus is shown in Fig. 1.

**TABLE 1.**
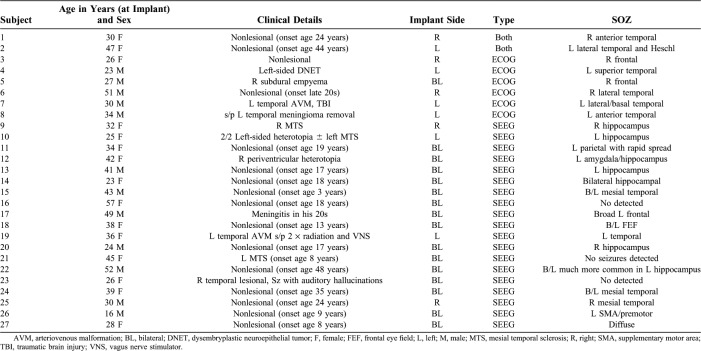
Demographic Characteristics of the Patients

### Correlation of Measures With Contact Depth

In the scenario where there are no statistically significant associations between the depth of the contact and quantitative measures of activity, we would expect that comparison of these measures would not yield many significant correlations. By contrast, if there is a systematic difference in activity with increasing contact depth, we would expect many significant correlations and for those correlations to have certain common characteristics such as different assumptions used in the analyses. Thus, the number of significant correlations with depth for quantitative measures of activity is a proxy for the tendency of the signal between ECOG and SEEG to systematically differ.

We performed a total of 303,552 correlations of activity with contact depth. These were performed using the four ways of measuring depth, two referencing schemes, and with and without baseline correction by Z-scoring. These multiple sets of assumptions were used to maximize the possibility that we would detect a systematic correlation of activity with increasing contact depth. As the number of significant correlations is determined by the type of multiple comparisons correction, we report for using different statistical thresholds. Using false discovery rate, 4,174 (1.3%) of the measures—either of spectral power or of PAC—were found to have a significant correlation with contact depth. Using Bonferroni correction, 51 (0.02%) of the measures—either of spectral power or of PAC—were found have a significant correlation with contact depth. These findings are summarized in Table 2.

**TABLE 2.**
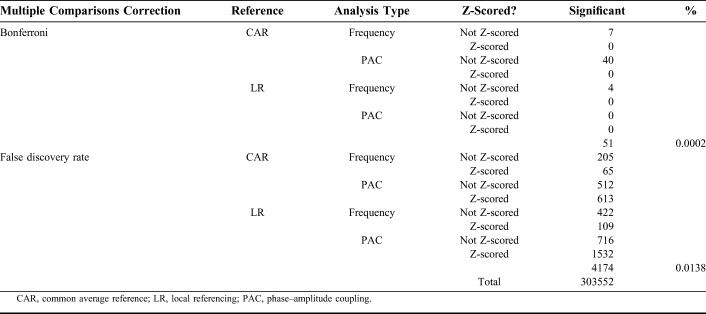
The Number of Statistically Significant Correlations (by Pearson *r*) with Contact Depth Using Different Sets of Assumptions (Referencing, Baseline Correction by Z-scoring) Under Different Methods of Multiple Comparisons Correction

### Characteristics of Statistically Significant Correlations With Contact Depth

**FIG. 2.**
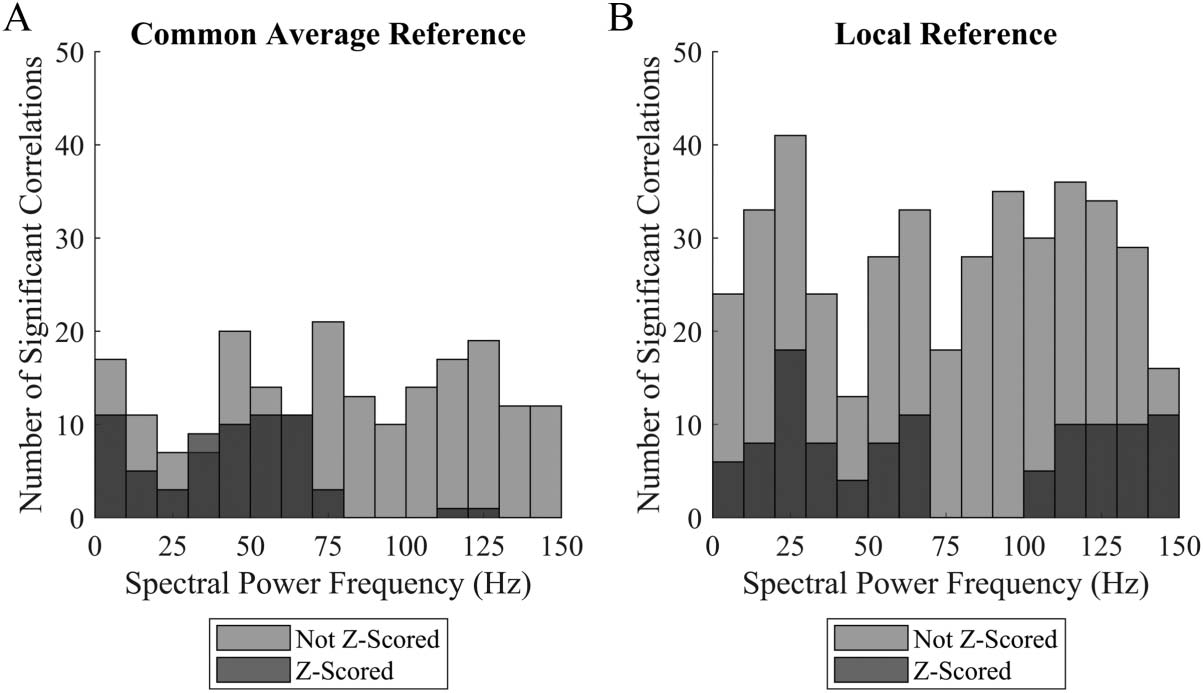
The number of significant correlations of spectral power with contact depth for CAR (**A**) and LR (**B**) referencing schemes are shown. The data are divided by the frequency of the spectral power. Z-scored spectrograms are indicated in blue; not Z-scored spectrograms are indicated in red. Note that there is a trend toward fewer significant correlations with depth—proxies for the difference between ECOG and SEEG activity—in the CAR and when the data are Z-scored. CAR, common average reference; ECOG, electrocorticography; LR, local referencing; SEEG, stereoelectroencephalography.

**FIG. 3.**
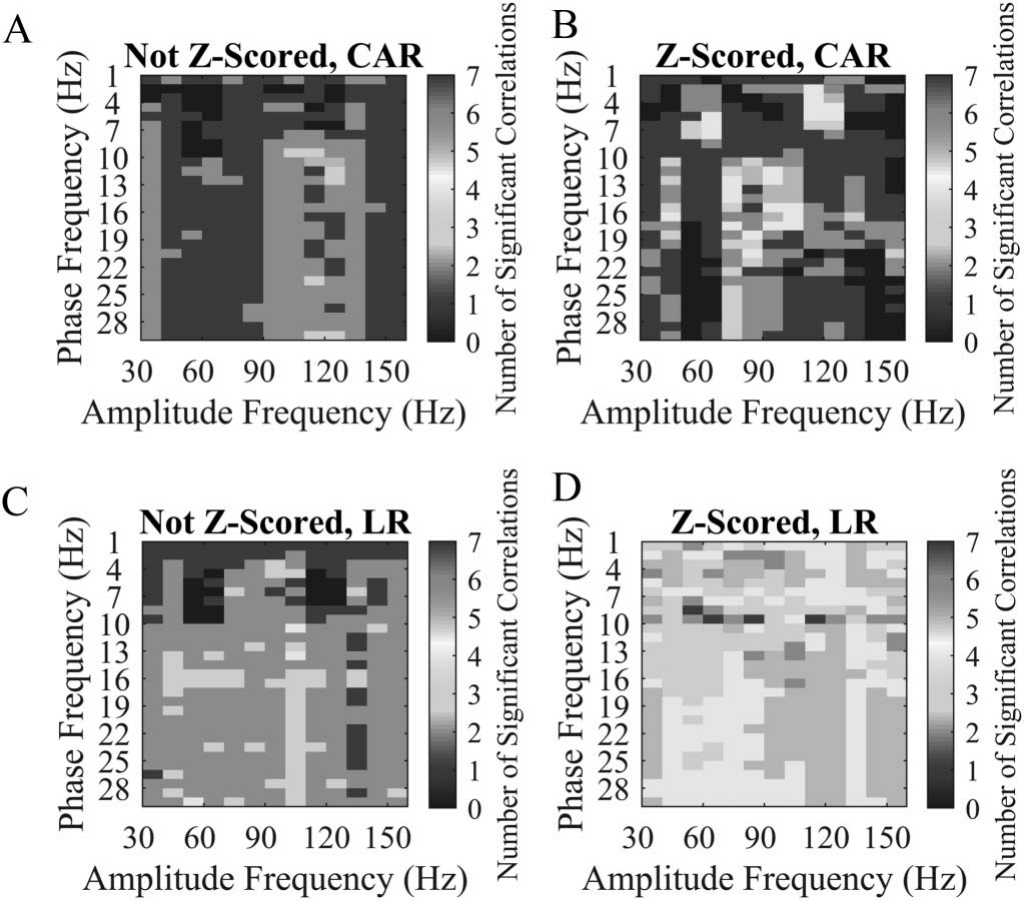
The number of significant correlations of PAC with contact depth for CAR (**A** and **B**) and LR (**C** and **D**) are shown. The data are divided by the frequency of the spectral power. Z-scored PAC values are indicated in (**B**) and (**D**); not Z-scored PAC values are indicated in (**A**) and (**C**). Warmer values indicate more significant correlations of PAC with contact depth. Note that there is a trend toward fewer significant correlations with depth—proxies for the difference between ECOG and SEEG activity—in CAR and when the data are not baseline corrected by Z-scoring. CAR, common average reference; ECOG, electrocorticography; LR, local referencing; PAC, phase–amplitude coupling; SEEG, stereoelectroencephalography.

For those measures where a statistically significant correlation with contact depth was observed, we attempted to determine whether there were any characteristics of the analysis that made it more likely to be significant. For subsequent analyses, we used multiple comparisons correction with FDR. With respect to spectral power, there was a trend toward fewer significant differences with depth in CAR versus LR (Fig. 2). Fewer differences were present when the spectral power was baseline corrected with Z-scoring, particularly at higher frequencies using CAR. This is likely because as the contact depth increases, there is frequently generalized attenuation of the signal leading to overall lower spectral power. By adjusting the baseline, Z-scoring removes this trend. With respect to PAC, there was again a trend toward fewer significant differences with depth in CAR versus LR (Fig. 3). By contrast, baseline correction by Z-scoring in the setting of PAC increased the number of significant differences. This is likely because the effect of attenuation of spectral power does not affect PAC calculations, as PAC calculates the relationship between the peaks of high frequencies and the phase of lower frequencies.

### Spatial Distribution of Statistically Significant Correlations with Contact Depth

**FIG. 4.**
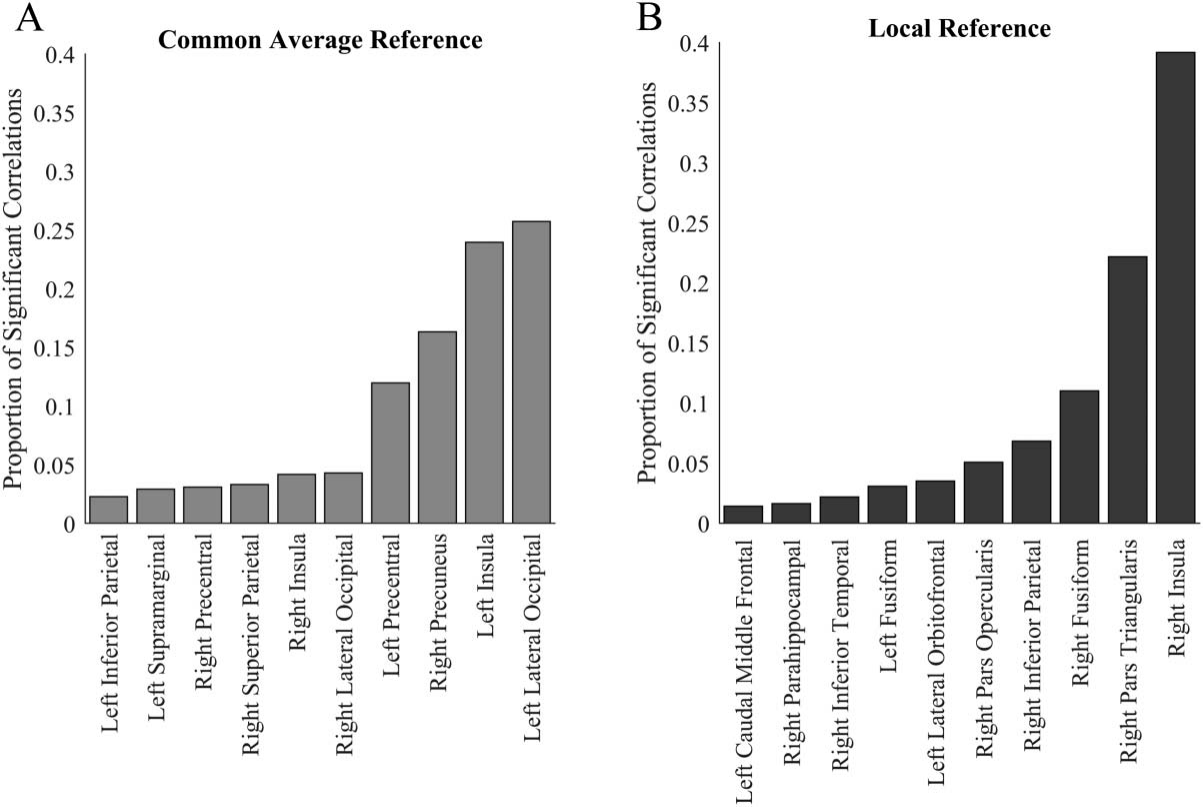
A histogram of the number of significant correlations of spectral power and PAC with contact depth for the 10 most prevalent brain regions, for CAR (**A**) and LR (**B**). Note the proportion of significant correlations tends to cluster in a couple of brain regions. CAR, common average reference; LR, local referencing; PAC, phase–amplitude coupling.

**FIG. 5.**
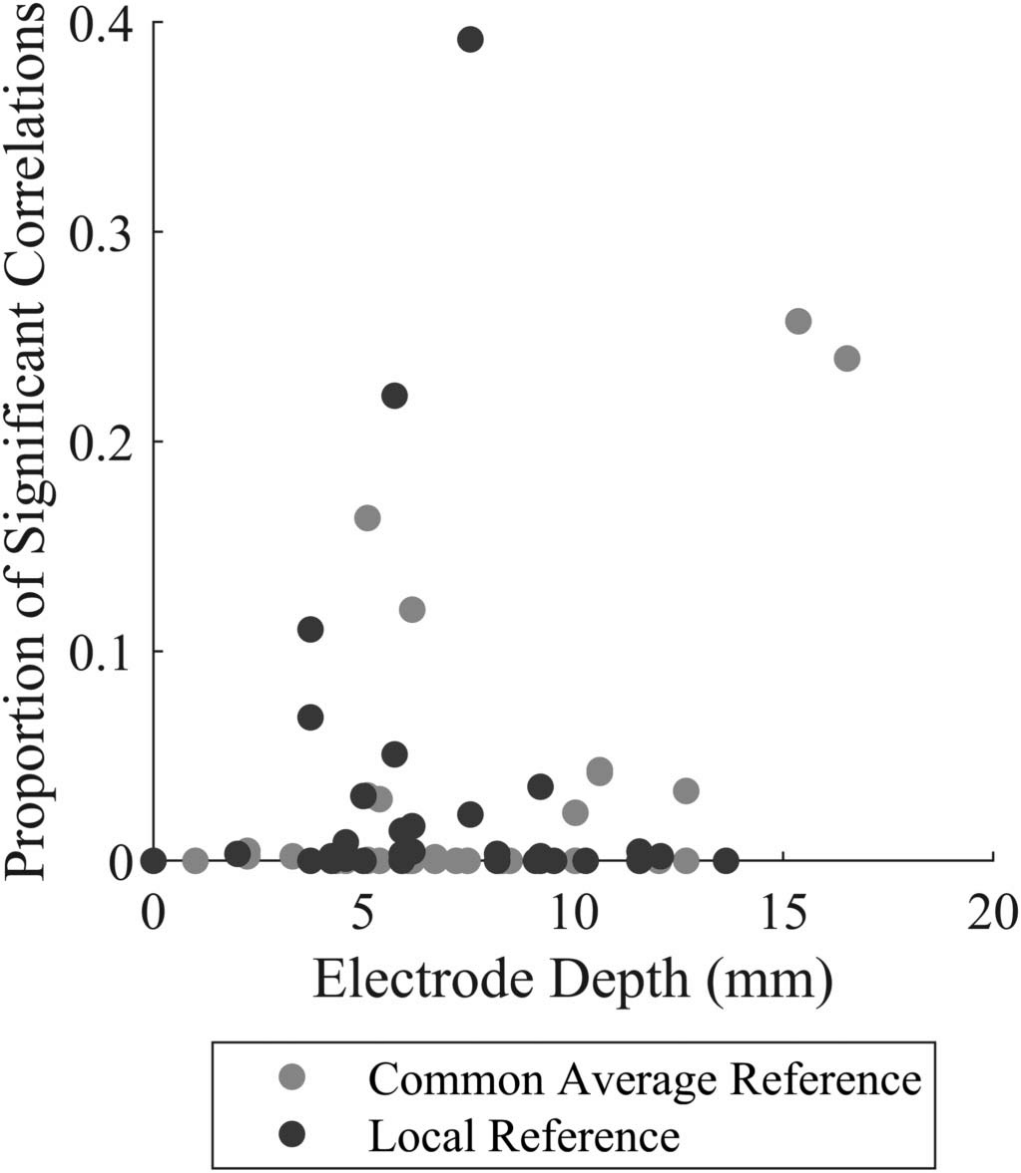
The association between the proportion of significant correlations of quantitative measures with contact depth with a brain region—a proxy for the difference between ECOG and SEEG signals—and the depth of the deepest contact in that brain region. Note that below 4 mm in maximum depth, very few significant correlations with depth are observed. Deeper than 4 mm, correlations of quantitative measures with depth are generally still low but can be much higher. ECOG, electrocorticography; SEEG, stereoelectroencephalography.

There was a nonrandom distribution of significant correlations with depth with many of the significant values concentrating in a few key regions (Fig. 4). For example, about 40% of the significant differences were found in the right insula using LR. Inspecting the spectrograms of these key regions suggests that one cause for this nonrandom distribution may be the depth of the contacts in that region. Figure 5 shows the relationship between the proportion of *significant correlations with depth*—a proxy for the difference in signal between ECOG and SEEG—and the deepest contact present in that region. When brain regions that have contacts no deeper than approximately 4 mm are considered, there are nearly no significant correlations with depth. Deeper than 4 mm, it is still likely that there will be no correlations with depth, but some brain regions begin to develop significant correlations.

**FIG. 6.**
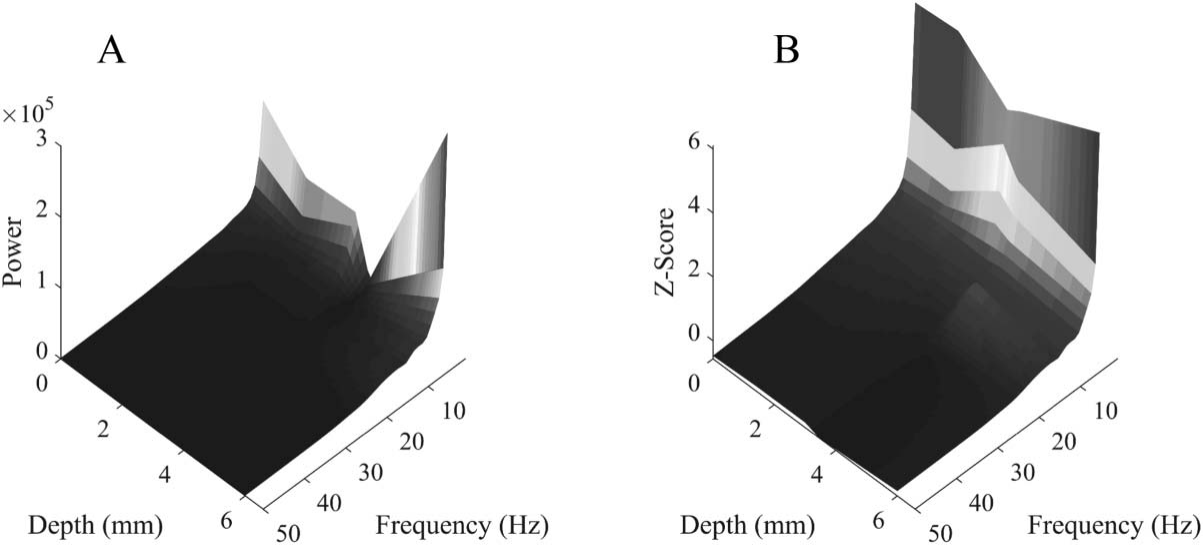
The spectral power (**A**) or Z-scored spectral power (**B**) in the right insula for all included contacts as a function of depth and frequency. Warmer colors indicate a higher spectral power or Z-scored spectral power. The data using LR are depicted. Note the presence of another spectral power source located at approximately 6 mm that is best observed in the not Z-scored data. LR, local referencing.

Figure 6 depicts an example of a brain region with deep sources that may contaminate the signal sensed by the contacts. For example, Fig. 6 shows the spectrograms for the right insula using the LR referencing scheme. The not-Z-scored data show the presence of a deep source that may increase spectral power or PAC higher than would be observed at that region. **Supplemental Digital Content 2** (see **Figure 1**, http://links.lww.com/JCNP/A56) shows the location of the deepest contact (from subject 11) that localized to the right insula. While the right insula was the closest grey matter voxel, the contact proximity to other deep and cortical structures may contaminate the signal recorded from that region. Hence, the presence of deep sources near a particular brain region may account for the nonrandom spatial distribution of significant correlations with contact depth.

### Statistically Significant Correlations With Contact Depth for the Two Subjects With Both Studies

Only two of the patients (subjects 1 and 2) had both SEEG and ECOG performed, so only these subjects could be directly compared. We performed a subset analysis including only these subjects that included 80,104 correlations of activity with contact depth. These were performed using the four ways of measuring depth, two referencing schemes, and with and without baseline correction by Z-scoring. By contrast, using false discovery rate or Bonferroni correction, none of the measures—either of spectral power or PAC—were found have a significant correlation with contact depth.

### Statistically Significant Correlations With Contact Depth Grouped by Bands

Because spectral power measures in 1 Hz increments may be highly correlated with neighboring increments, we performed 3,392 additional correlations with electrode depth, grouping spectral power into bands—delta (1–3 Hz), theta (4–7 Hz), alpha (8–14 Hz), beta (15–29 Hz), low gamma (30–79 Hz), and high gamma (80–150 Hz)—as well as broad band spectral power (1–150 Hz) and broad band PAC (1–20 Hz phase frequency, 30–150 Hz amplitude frequency). These were performed using multiple approaches, i.e., four ways of measuring depth, two referencing schemes, and with and without baseline correction by Z-scoring. Using false discovery rate, 34 (1%) of the measures—either of spectral power or PAC—were found have a significant correlation with contact depth. Using Bonferroni correction, 9 (0.003%) of the measures—either of spectral power or PAC—were found have a significant correlation with contact depth. These findings are using FDR for the LR scheme in **Supplemental Digital Content 2** (see, **Figure 2**, http://links.lww.com/JCNP/A56). Note that there were no significant correlations using CAR.

## DISCUSSION

This study compiled numerical measures of spectral power and PAC from invasive recording studies, SEEG, ECOG, or both. We then examined the results of these measures to determine what, if any, of the measures are correlated with contact depth. Correlation of an activity measure with contact depth is used to represent the level of difference in signal, by quantitative measures, between SEEG and ECOG contacts. We repeated the analysis using multiple sets of assumptions, including ways of measuring depth, referencing schemes, and baseline correction by Z-scoring, to determine whether any observed difference was associated with a particular set of analytic assumptions.

Using even the most permissive possible statistical criterion, our results demonstrate that differences between SEEG and ECOG signals are not strongly correlated with contact depth. There was a limited correlation with electrode depth even when the subset of subjects with both SEEG and ECOG were considered. These results support the idea that SEEG may be substituted for ECOG under most circumstances and may provide similar signals.

Our data also provide technical guidance for the circumstance under which the two modalities may provide divergent information. The characteristics that were associated with divergence between the two modalities were as follows: (1) failing to baseline correct to account for generalized attenuation in the setting of increasing contact depth in the setting of spectral power analyses; (2) the use of a local rather than a common average reference; (3) using baseline correction in the setting of PAC calculations; and (4) inclusion of contacts that are potentially in proximity to other gray and white matter sources outside the region of interest or, in our data, contact depth greater than 4 mm. The identification of these factors allows clinicians and scientists to identify the circumstances under which caution should be used in treating signals from SEEG and ECOG as equivalent.

Our data show some discrepancy with the existing literature with respect to referencing. We show that there is a trend toward increasing divergence between SEEG and ECOG with the use of a LR versus CAR. Others have shown that the use of a LR rather than a CAR lowers the correlation between contacts and limits the effect of volume conduction.^33^ Our data appear to support this finding by showing that there is a greater discrepancy between contacts on the basis of depth in the setting of LR, whereas this discrepancy is smaller when using CAR. It is important to note, however, that the previous study evaluated correlation, whereas this study focuses on spectral power and PAC. It may be that measures of connectivity such as correlation are more sensitive to referencing or require different referencing schemes than spectral power and PAC.

We acknowledge several caveats that limit our study generalizability. First, similar to previous studies cataloguing intracranial activity,^18^ the proportion of ECOG contacts was much lower than SEEG contacts. This study used approximately 20% ECOG contacts in the data set. It is possible that a higher concentration of ECOG contacts would show a greater divergence between the modalities. Second, because of clinical considerations and the desire for a broader data set, the behavior of the subjects during the pruned interval is heterogeneous. It is possible that there would be greater divergence between the modalities if the subjects were all performing a specific cognitive task. Finally, this study focused on quantitative measures of intracranial activity, including spectral power and PAC. We cannot comment on other measures of the signal such as measures of connectivity or waveform shape. It may be that these measures differ more widely between ECOG and SEEG. Finally, only two of the subjects in this study had both studies performed. Given interindividual variation in activity, a large number of subjects who underwent both studies may be required to determine systematic differences between the studies.

This study compared the quantitative analysis of intracranial EEG signals using either SEEG or ECOG studies. We show that the two modalities appear to be similar. However, factors that are associated with differences between the modalities include the use baseline correction by Z-scoring, the choice of reference, and the presence of other signal sources near the contacts. These results provide technical guidance for when SEEG may be substituted for ECOG in a clinical or scientific setting, though the findings may not be generalizable and should be replicated in a larger population. Nevertheless, our data provide reassurance that electroclinical data from both modalities are comparable and that the advantageous breadth and depth of SEEG monitoring over subdural grids, combined with its less invasive application, will lead to more widespread application of SEEG in the evaluation of epilepsy.

## Supplementary Material

SUPPLEMENTARY MATERIAL
