# Supplementary figures and images for: Quantitative Signal Characteristics of Electrocorticography and Stereoelectroencephalography: The Effect of Contact Depth
Source: J Clin Neurophysiol. 2019 Mar 26;36(3):195–203. doi: 10.1097/WNP.0000000000000577 (PMC6493682; doi:10.1097/WNP.0000000000000577)

## Slide 1
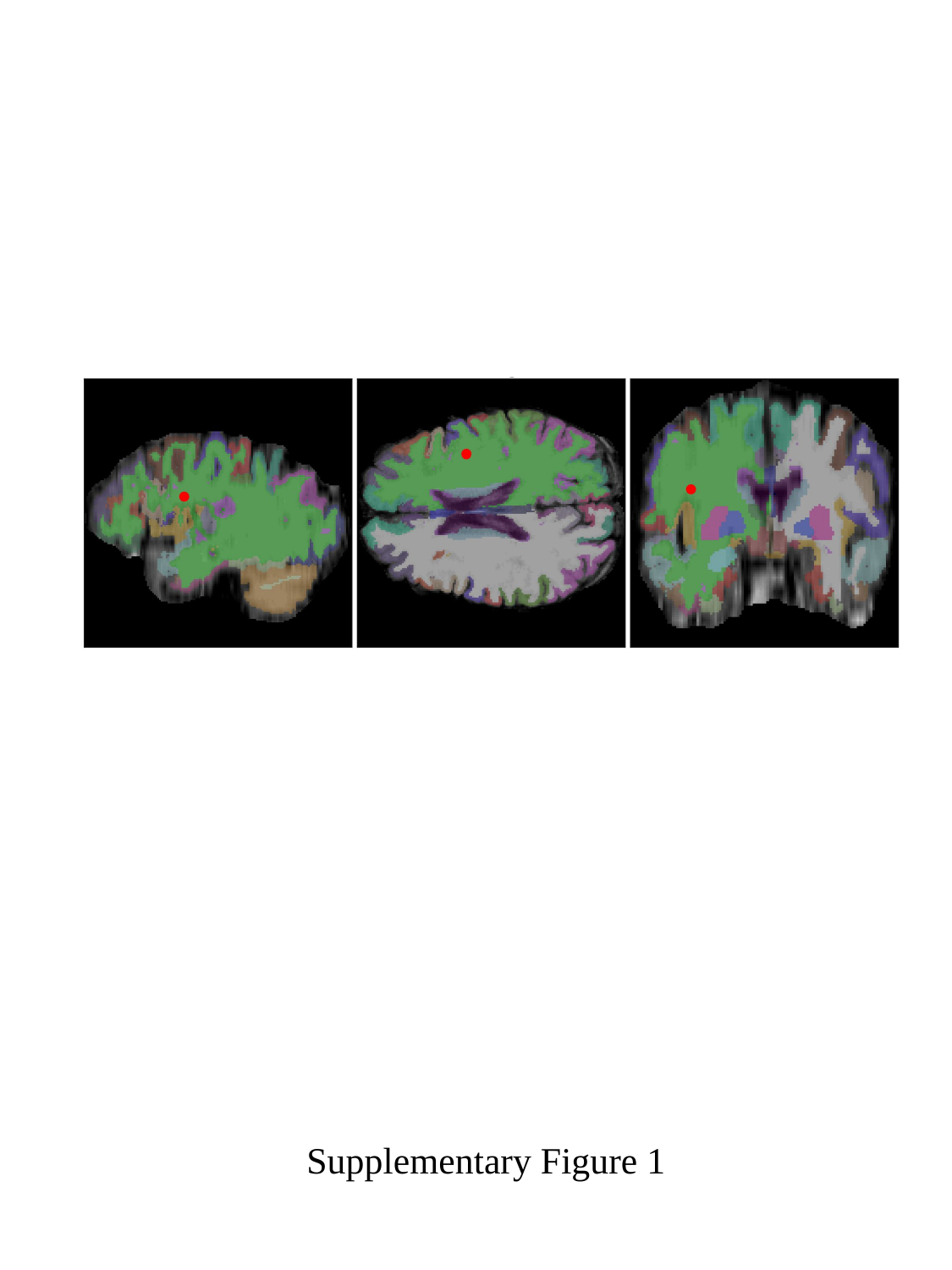

Supplementary Figure 1

## Slide 2
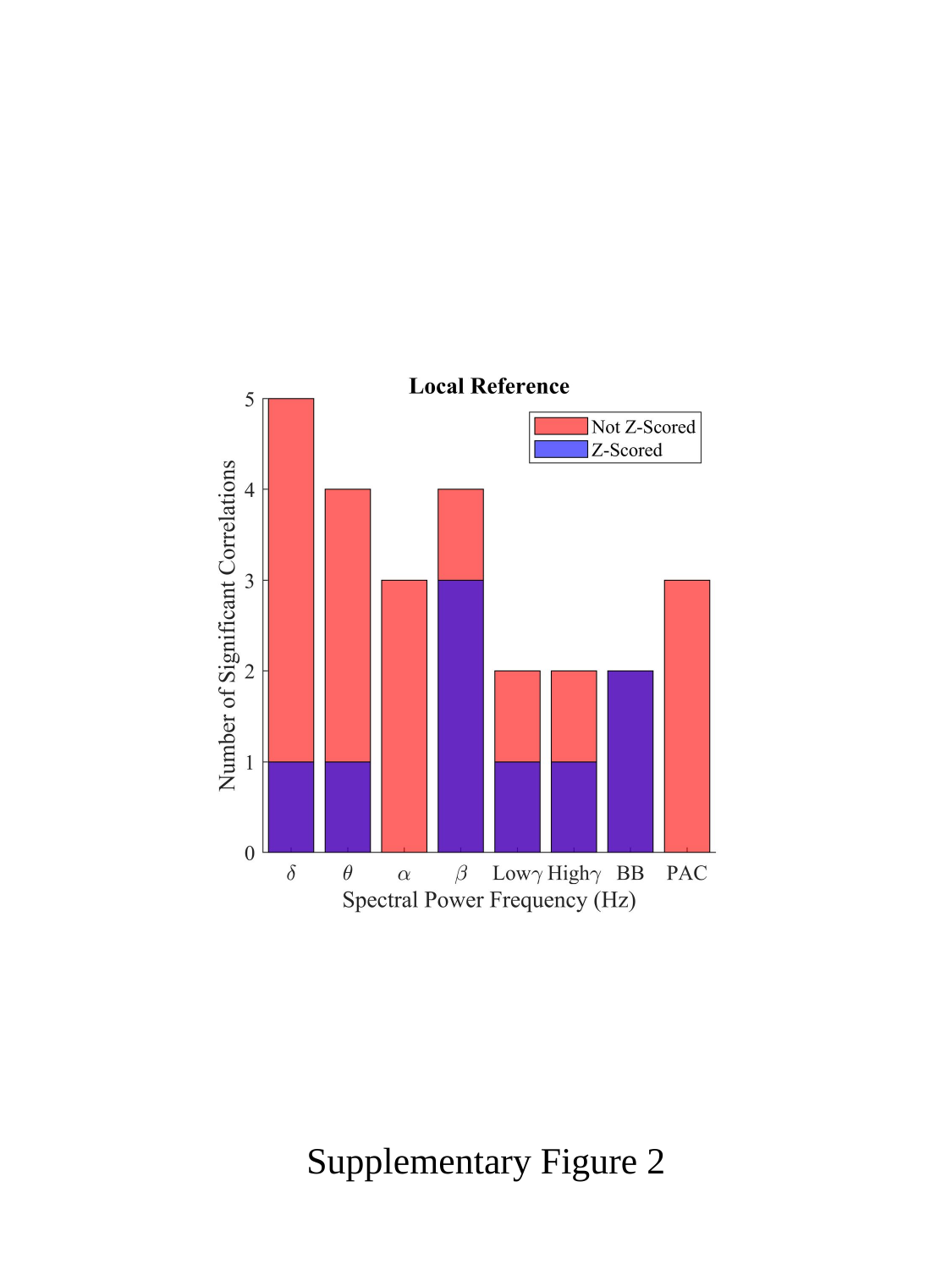

Supplementary Figure 2

Supplement: SUPPLEMENTARY MATERIAL [file jcnp-36-195-s001.pptx]
