# Supplementary material for: Quantitative Signal Characteristics of Electrocorticography and Stereoelectroencephalography: The Effect of Contact Depth
Source: J Clin Neurophysiol. 2019 Mar 26;36(3):195–203. doi: 10.1097/WNP.0000000000000577 (PMC6493682; doi:10.1097/WNP.0000000000000577)
Supplement: SUPPLEMENTARY MATERIAL [file jcnp-36-195-s002.doc]

**CAR**

| **Location** | **Subject** | **VMD** | **Type** |
| --- | --- | --- | --- |
| Right Banks of the Superior Temporal Sulcus | 1 | 0 | Grid |
| Right Banks of the Superior Temporal Sulcus | 1 | 7.2111 | Depth |
| Right Caudal Middle Frontal | 1 | 0 | Grid |
| Right Entorhinal | 1 | 0 | Strip |
| Right Frontal Pole | 1 | 0 | Strip |
| Right Fusiform | 1 | 0 | Strip |
| Right Inferior Parietal | 1 | 0 | Grid |
| Right Inferior Parietal | 1 | 0 | SEEG |
| Right Inferior Parietal | 1 | 1 | SEEG |
| Right Inferior Parietal | 1 | 3.6056 | SEEG |
| Right Inferior Parietal | 1 | 4.1231 | SEEG |
| Right Inferior Parietal | 1 | 5.4772 | SEEG |
| Right Inferior Parietal | 1 | 6.4031 | SEEG |
| Right Inferior Parietal | 1 | 8.4853 | SEEG |
| Right Inferior Temporal | 1 | 0 | Grid |
| Right Inferior Temporal | 1 | 1 | SEEG |
| Right Inferior Temporal | 1 | 2.8284 | SEEG |
| Right Inferior Temporal | 1 | 8.6023 | SEEG |
| Right Inferior Temporal | 1 | 9.2195 | SEEG |
| Right Insula | 1 | 0 | Grid |
| Right Lateral Occipital | 1 | 0 | Grid |
| Right Lateral Orbitofrontal | 1 | 0 | Strip |
| Right Lateral Orbitofrontal | 1 | 1 | SEEG |
| Right Lateral Orbitofrontal | 1 | 4 | SEEG |
| Right Middle Temporal | 1 | 0 | Grid |
| Right Middle Temporal | 1 | 1.4142 | SEEG |
| Right Middle Temporal | 1 | 3.7417 | SEEG |
| Right Parahippocampal | 1 | 0 | Strip |
| Right Pars Opercularis | 1 | 0 | Grid |
| Right Pars Triangularis | 1 | 0 | Grid |
| Right Pars Triangularis | 1 | 2 | SEEG |
| Right Pars Triangularis | 1 | 3.1623 | SEEG |
| Right Postcentral | 1 | 0 | Grid |
| Right Precentral | 1 | 0 | Grid |
| Right Precuneus | 1 | 6.4031 | SEEG |
| Right Precuneus | 1 | 9.434 | SEEG |
| Right Rostral Middle Frontal | 1 | 0 | Grid |
| Right Superior Parietal | 1 | 0 | Grid |
| Right Superior Parietal | 1 | 0 | SEEG |
| Right Superior Parietal | 1 | 6.7082 | SEEG |
| Right Superior Parietal | 1 | 7.0711 | SEEG |
| Right Superior Parietal | 1 | 12.083 | SEEG |
| Right Superior Temporal | 1 | 0 | Grid |
| Right Supramarginal | 1 | 0 | Grid |
| Right Supramarginal | 1 | 3.7417 | Depth |
| Left Banks of the Superior Temporal Sulcus | 2 | 0 | SEEG |
| Left Banks of the Superior Temporal Sulcus | 2 | 1 | SEEG |
| Left Banks of the Superior Temporal Sulcus | 2 | 6.1644 | SEEG |
| Left Banks of the Superior Temporal Sulcus | 2 | 0 | Grid |
| Left Caudal Middle Frontal | 2 | 0 | Grid |
| Left Entorhinal | 2 | 0 | Strip |
| Left Fusiform | 2 | 2.2361 | SEEG |
| Left Fusiform | 2 | 4.1231 | SEEG |
| Left Fusiform | 2 | 4.899 | SEEG |
| Left Fusiform | 2 | 6.1644 | SEEG |
| Left Fusiform | 2 | 0 | Strip |
| Left Inferior Parietal | 2 | 1 | SEEG |
| Left Inferior Parietal | 2 | 2.4495 | SEEG |
| Left Inferior Parietal | 2 | 3.1623 | SEEG |
| Left Inferior Temporal | 2 | 2.2361 | SEEG |
| Left Inferior Temporal | 2 | 3.7417 | SEEG |
| Left Inferior Temporal | 2 | 4.1231 | SEEG |
| Left Inferior Temporal | 2 | 0 | Strip |
| Left Insula | 2 | 0 | Grid |
| Left Lateral Orbitofrontal | 2 | 1 | SEEG |
| Left Lateral Orbitofrontal | 2 | 0 | Strip |
| Left Middle Temporal | 2 | 0 | SEEG |
| Left Middle Temporal | 2 | 0 | Grid |
| Left Pars Opercularis | 2 | 0 | Grid |
| Left Pars Orbitalis | 2 | 2.2361 | SEEG |
| Left Pars Orbitalis | 2 | 2.8284 | SEEG |
| Left Pars Orbitalis | 2 | 0 | Grid |
| Left Pars Triangularis | 2 | 1.4142 | SEEG |
| Left Pars Triangularis | 2 | 2 | SEEG |
| Left Pars Triangularis | 2 | 2.8284 | SEEG |
| Left Pars Triangularis | 2 | 3 | SEEG |
| Left Pars Triangularis | 2 | 0 | Grid |
| Left Postcentral | 2 | 11.5758 | SEEG |
| Left Postcentral | 2 | 0 | Grid |
| Left Precentral | 2 | 6.4031 | SEEG |
| Left Precentral | 2 | 0 | Grid |
| Left Rostral Middle Frontal | 2 | 0 | SEEG |
| Left Rostral Middle Frontal | 2 | 1 | SEEG |
| Left Rostral Middle Frontal | 2 | 1.4142 | SEEG |
| Left Rostral Middle Frontal | 2 | 0 | Grid |
| Left Superior Temporal | 2 | 8.1854 | SEEG |
| Left Superior Temporal | 2 | 0 | Grid |
| Left Supramarginal | 2 | 4.2426 | SEEG |
| Left Supramarginal | 2 | 0 | Grid |
| Left Temporal Pole | 2 | 0 | Grid |
| Right Caudal Middle Frontal | 3 | 0 | Grid |
| Right Entorhinal | 3 | 0 | Strip |
| Right Fusiform | 3 | 0 | Strip |
| Right Inferior Parietal | 3 | 0 | Grid |
| Right Inferior Temporal | 3 | 0 | Strip |
| Right Inferior Temporal | 3 | 8.544 | Depth |
| Right Insula | 3 | 0 | Grid |
| Right Lateral Occipital | 3 | 0 | Strip |
| Right Middle Temporal | 3 | 0 | Strip |
| Right Middle Temporal | 3 | 12.6886 | Depth |
| Right Pars Opercularis | 3 | 0 | Grid |
| Right Postcentral | 3 | 0 | Grid |
| Right Precentral | 3 | 0 | Grid |
| Right Superior Frontal | 3 | 0 | Grid |
| Right Superior Parietal | 3 | 0 | Grid |
| Right Superior Temporal | 3 | 0 | Strip |
| Right Superior Temporal | 3 | 5.7446 | Depth |
| Right Supramarginal | 3 | 0 | Grid |
| Right Temporal Pole | 3 | 0 | Strip |
| Right Transverse Temporal | 3 | 12.083 | Depth |
| Right Banks of the Superior Temporal Sulcus | 4 | 0 | Grid |
| Right Entorhinal | 4 | 0 | Strip |
| Right Fusiform | 4 | 0 | Strip |
| Right Inferior Parietal | 4 | 0 | Grid |
| Right Inferior Temporal | 4 | 0 | Strip |
| Right Lateral Occipital | 4 | 0 | Grid |
| Right Middle Temporal | 4 | 0 | Grid |
| Right Parahippocampal | 4 | 0 | Strip |
| Right Pars Opercularis | 4 | 0 | Grid |
| Right Pars Triangularis | 4 | 0 | Grid |
| Right Postcentral | 4 | 0 | Grid |
| Right Precentral | 4 | 0 | Grid |
| Right Rostral Middle Frontal | 4 | 0 | Grid |
| Right Superior Parietal | 4 | 0 | Grid |
| Right Superior Temporal | 4 | 0 | Grid |
| Right Supramarginal | 4 | 0 | Grid |
| Right Temporal Pole | 4 | 0 | Grid |
| Right Transverse Temporal | 4 | 7.874 | Depth |
| Right Transverse Temporal | 4 | 9.2736 | Depth |
| Right Banks of the Superior Temporal Sulcus | 5 | 0 | Grid |
| Right Caudal Middle Frontal | 5 | 0 | Grid |
| Right Fusiform | 5 | 0 | Strip |
| Right Inferior Parietal | 5 | 0 | Grid |
| Right Inferior Temporal | 5 | 0 | Strip |
| Right Middle Temporal | 5 | 0 | Grid |
| Right Pars Triangularis | 5 | 0 | Grid |
| Right Postcentral | 5 | 0 | Grid |
| Right Precuneus | 5 | 0 | Strip |
| Right Superior Frontal | 5 | 0 | Grid |
| Right Superior Parietal | 5 | 0 | Grid |
| Right Supramarginal | 5 | 0 | Grid |
| Right Entorhinal | 6 | 0 | Strip |
| Right Frontal Pole | 6 | 0 | Strip |
| Right Fusiform | 6 | 0 | Strip |
| Right Inferior Parietal | 6 | 0 | Grid |
| Right Inferior Temporal | 6 | 0 | Strip |
| Right Lateral Occipital | 6 | 0 | Grid |
| Right Lateral Orbitofrontal | 6 | 0 | Grid |
| Right Medial Orbitofrontal | 6 | 0 | Strip |
| Right Middle Temporal | 6 | 0 | Grid |
| Right Parahippocampal | 6 | 0 | Strip |
| Right Pars Opercularis | 6 | 0 | Grid |
| Right Pars Orbitalis | 6 | 0 | Grid |
| Right Pars Triangularis | 6 | 0 | Grid |
| Right Postcentral | 6 | 0 | Grid |
| Right Precentral | 6 | 0 | Grid |
| Right Rostral Middle Frontal | 6 | 0 | Grid |
| Right Superior Temporal | 6 | 0 | Grid |
| Right Supramarginal | 6 | 0 | Grid |
| Right Temporal Pole | 6 | 0 | Grid |
| Right Transverse Temporal | 6 | 0 | Grid |
| Left Caudal Middle Frontal | 7 | 0 | Grid |
| Left Fusiform | 7 | 0 | Grid |
| Left Inferior Parietal | 7 | 0 | Grid |
| Left Inferior Temporal | 7 | 0 | Grid |
| Left Lateral Occipital | 7 | 0 | Grid |
| Left Middle Temporal | 7 | 0 | Grid |
| Left Pars Opercularis | 7 | 0 | Grid |
| Left Pars Orbitalis | 7 | 0 | Grid |
| Left Pars Triangularis | 7 | 0 | Grid |
| Left Postcentral | 7 | 0 | Grid |
| Left Precentral | 7 | 0 | Grid |
| Left Rostral Middle Frontal | 7 | 0 | Grid |
| Left Superior Temporal | 7 | 0 | Grid |
| Left Supramarginal | 7 | 0 | Grid |
| Left Caudal Middle Frontal | 8 | 0 | Grid |
| Left Inferior Temporal | 8 | 0 | Strip |
| Left Middle Temporal | 8 | 0 | Strip |
| Left Pars Opercularis | 8 | 0 | Grid |
| Left Pars Orbitalis | 8 | 0 | Grid |
| Left Pars Triangularis | 8 | 0 | Grid |
| Left Postcentral | 8 | 0 | Grid |
| Left Precentral | 8 | 0 | Grid |
| Left Rostral Middle Frontal | 8 | 0 | Grid |
| Left Superior Temporal | 8 | 0 | Grid |
| Left Supramarginal | 8 | 0 | Grid |
| Left Transverse Temporal | 8 | 0 | Grid |
| Right Middle Temporal | 9 | 2.4495 | SEEG |
| Right Superior Temporal | 9 | 1 | SEEG |
| Left Caudal Middle Frontal | 10 | 3.1623 | SEEG |
| Left Caudal Middle Frontal | 10 | 4.1231 | SEEG |
| Left Caudal Middle Frontal | 10 | 6.7082 | SEEG |
| Left Fusiform | 10 | 7.3485 | SEEG |
| Left Inferior Parietal | 10 | 4.2426 | SEEG |
| Left Lateral Orbitofrontal | 10 | 4.3589 | SEEG |
| Left Middle Temporal | 10 | 1 | SEEG |
| Left Middle Temporal | 10 | 1.4142 | SEEG |
| Left Middle Temporal | 10 | 3.6056 | SEEG |
| Left Middle Temporal | 10 | 4.5826 | SEEG |
| Left Middle Temporal | 10 | 5 | SEEG |
| Left Middle Temporal | 10 | 5.3852 | SEEG |
| Left Pars Orbitalis | 10 | 0 | SEEG |
| Left Caudal Middle Frontal | 11 | 0 | SEEG |
| Left Caudal Middle Frontal | 11 | 1.4142 | SEEG |
| Left Caudal Middle Frontal | 11 | 2.2361 | SEEG |
| Left Caudal Middle Frontal | 11 | 2.4495 | SEEG |
| Left Caudal Middle Frontal | 11 | 3.1623 | SEEG |
| Left Caudal Middle Frontal | 11 | 3.3166 | SEEG |
| Left Caudal Middle Frontal | 11 | 4.1231 | SEEG |
| Left Fusiform | 11 | 0 | SEEG |
| Left Inferior Parietal | 11 | 1.4142 | SEEG |
| Left Inferior Parietal | 11 | 2.2361 | SEEG |
| Left Inferior Parietal | 11 | 3 | SEEG |
| Left Inferior Temporal | 11 | 1 | SEEG |
| Left Inferior Temporal | 11 | 2 | SEEG |
| Left Insula | 11 | 1 | SEEG |
| Left Insula | 11 | 1.4142 | SEEG |
| Left Insula | 11 | 1.7321 | SEEG |
| Left Insula | 11 | 2 | SEEG |
| Left Insula | 11 | 4.899 | SEEG |
| Left Precentral | 11 | 5 | SEEG |
| Left Precentral | 11 | 8.1854 | SEEG |
| Left Precentral | 11 | 9.2195 | SEEG |
| Left Rostral Middle Frontal | 11 | 3.1623 | SEEG |
| Left Superior Temporal | 11 | 2.2361 | SEEG |
| Right Caudal Middle Frontal | 11 | 0 | SEEG |
| Right Caudal Middle Frontal | 11 | 1 | SEEG |
| Right Caudal Middle Frontal | 11 | 2.4495 | SEEG |
| Right Caudal Middle Frontal | 11 | 3.1623 | SEEG |
| Right Inferior Parietal | 11 | 0 | SEEG |
| Right Inferior Parietal | 11 | 1 | SEEG |
| Right Inferior Parietal | 11 | 5.7446 | SEEG |
| Right Insula | 11 | 1.4142 | SEEG |
| Right Insula | 11 | 6.0828 | SEEG |
| Right Middle Temporal | 11 | 2.4495 | SEEG |
| Right Precentral | 11 | 1 | SEEG |
| Right Precentral | 11 | 3 | SEEG |
| Right Precentral | 11 | 4.2426 | SEEG |
| Right Precentral | 11 | 4.3589 | SEEG |
| Right Superior Parietal | 11 | 1.7321 | SEEG |
| Right Superior Parietal | 11 | 4.2426 | SEEG |
| Right Superior Temporal | 11 | 0 | SEEG |
| Right Transverse Temporal | 11 | 2 | SEEG |
| Left Fusiform | 12 | 3.4641 | SEEG |
| Left Fusiform | 12 | 7.8102 | SEEG |
| Left Fusiform | 12 | 9.6437 | SEEG |
| Left Inferior Parietal | 12 | 8.6023 | SEEG |
| Left Inferior Parietal | 12 | 10.198 | SEEG |
| Left Inferior Parietal | 12 | 14.1774 | SEEG |
| Left Inferior Parietal | 12 | 15.3623 | SEEG |
| Left Inferior Parietal | 12 | 16.5227 | SEEG |
| Right Superior Parietal | 12 | 13.6382 | SEEG |
| Right Superior Temporal | 12 | 4.1231 | SEEG |
| Right Superior Temporal | 12 | 5 | SEEG |
| Right Superior Temporal | 12 | 8.9443 | SEEG |
| Right Superior Temporal | 12 | 10.2956 | SEEG |
| Right Transverse Temporal | 12 | 2.8284 | SEEG |
| Left Fusiform | 13 | 2.2361 | SEEG |
| Left Fusiform | 13 | 3 | SEEG |
| Left Fusiform | 13 | 3.1623 | SEEG |
| Left Fusiform | 13 | 3.7417 | SEEG |
| Left Inferior Temporal | 13 | 3 | SEEG |
| Left Inferior Temporal | 13 | 5.099 | SEEG |
| Left Inferior Temporal | 13 | 6.4031 | SEEG |
| Left Insula | 13 | 5.7446 | SEEG |
| Left Insula | 13 | 6 | SEEG |
| Left Middle Temporal | 13 | 5.099 | SEEG |
| Left Middle Temporal | 13 | 5.3852 | SEEG |
| Left Rostral Middle Frontal | 13 | 1.7321 | SEEG |
| Left Rostral Middle Frontal | 13 | 2 | SEEG |
| Left Rostral Middle Frontal | 13 | 3 | SEEG |
| Left Rostral Middle Frontal | 13 | 4.1231 | SEEG |
| Left Superior Temporal | 13 | 1.4142 | SEEG |
| Left Superior Temporal | 13 | 2.2361 | SEEG |
| Left Superior Temporal | 13 | 2.8284 | SEEG |
| Right Fusiform | 13 | 2.2361 | SEEG |
| Right Inferior Temporal | 13 | 2 | SEEG |
| Right Inferior Temporal | 13 | 2.4495 | SEEG |
| Right Inferior Temporal | 13 | 5.7446 | SEEG |
| Right Inferior Temporal | 13 | 6.1644 | SEEG |
| Right Inferior Temporal | 13 | 8.0623 | SEEG |
| Right Middle Temporal | 13 | 3 | SEEG |
| Right Middle Temporal | 13 | 4.3589 | SEEG |
| Right Middle Temporal | 13 | 6.0828 | SEEG |
| Right Pars Triangularis | 13 | 1 | SEEG |
| Right Pars Triangularis | 13 | 4.5826 | SEEG |
| Right Rostral Middle Frontal | 13 | 4.4721 | SEEG |
| Left Inferior Temporal | 14 | 1 | SEEG |
| Left Insula | 14 | 2 | SEEG |
| Left Insula | 14 | 2.4495 | SEEG |
| Left Lateral Orbitofrontal | 14 | 2.2361 | SEEG |
| Left Middle Temporal | 14 | 1.7321 | SEEG |
| Left Middle Temporal | 14 | 3.7417 | SEEG |
| Left Pars Triangularis | 14 | 2.2361 | SEEG |
| Left Superior Temporal | 14 | 2.2361 | SEEG |
| Right Inferior Temporal | 14 | 1 | SEEG |
| Right Middle Temporal | 14 | 1.7321 | SEEG |
| Right Middle Temporal | 14 | 3 | SEEG |
| Right Middle Temporal | 14 | 4.1231 | SEEG |
| Right Pars Opercularis | 14 | 3.3166 | SEEG |
| Right Pars Triangularis | 14 | 1.7321 | SEEG |
| Right Pars Triangularis | 14 | 3 | SEEG |
| Right Rostral Middle Frontal | 14 | 0 | SEEG |
| Right Rostral Middle Frontal | 14 | 1 | SEEG |
| Right Rostral Middle Frontal | 14 | 1.7321 | SEEG |
| Right Superior Frontal | 14 | 1.4142 | SEEG |
| Right Superior Frontal | 14 | 2 | SEEG |
| Right Superior Temporal | 14 | 0 | SEEG |
| Right Superior Temporal | 14 | 1.7321 | SEEG |
| Right Superior Temporal | 14 | 2.8284 | SEEG |
| Left Caudal Middle Frontal | 15 | 4.4721 | SEEG |
| Left Fusiform | 15 | 3.4641 | SEEG |
| Left Inferior Temporal | 15 | 1.4142 | SEEG |
| Left Inferior Temporal | 15 | 3 | SEEG |
| Left Inferior Temporal | 15 | 4.2426 | SEEG |
| Left Inferior Temporal | 15 | 4.899 | SEEG |
| Left Insula | 15 | 1 | SEEG |
| Left Middle Temporal | 15 | 3.7417 | SEEG |
| Left Middle Temporal | 15 | 4.5826 | SEEG |
| Left Pars Opercularis | 15 | 5.3852 | SEEG |
| Left Precentral | 15 | 2.2361 | SEEG |
| Left Rostral Middle Frontal | 15 | 2.8284 | SEEG |
| Left Rostral Middle Frontal | 15 | 3 | SEEG |
| Left Rostral Middle Frontal | 15 | 4 | SEEG |
| Right Fusiform | 15 | 3.1623 | SEEG |
| Right Inferior Parietal | 15 | 0 | SEEG |
| Right Inferior Parietal | 15 | 2.4495 | SEEG |
| Right Inferior Parietal | 15 | 5.6569 | SEEG |
| Right Inferior Temporal | 15 | 2.4495 | SEEG |
| Right Inferior Temporal | 15 | 3 | SEEG |
| Right Inferior Temporal | 15 | 4.1231 | SEEG |
| Right Insula | 15 | 1 | SEEG |
| Right Insula | 15 | 1.4142 | SEEG |
| Right Insula | 15 | 3 | SEEG |
| Right Lateral Occipital | 15 | 2.2361 | SEEG |
| Right Lateral Orbitofrontal | 15 | 1.7321 | SEEG |
| Right Medial Orbitofrontal | 15 | 2.2361 | SEEG |
| Right Medial Orbitofrontal | 15 | 4.1231 | SEEG |
| Right Middle Temporal | 15 | 1.4142 | SEEG |
| Right Middle Temporal | 15 | 2 | SEEG |
| Right Pars Triangularis | 15 | 2.2361 | SEEG |
| Right Precuneus | 15 | 2.2361 | SEEG |
| Right Precuneus | 15 | 4.5826 | SEEG |
| Right Precuneus | 15 | 9.5394 | SEEG |
| Right Rostral Middle Frontal | 15 | 1.4142 | SEEG |
| Right Rostral Middle Frontal | 15 | 5.3852 | SEEG |
| Right Rostral Middle Frontal | 15 | 6.1644 | SEEG |
| Right Superior Frontal | 15 | 2 | SEEG |
| Right Superior Frontal | 15 | 2.8284 | SEEG |
| Right Superior Frontal | 15 | 3.1623 | SEEG |
| Right Superior Frontal | 15 | 3.6056 | SEEG |
| Right Superior Frontal | 15 | 4.1231 | SEEG |
| Right Superior Parietal | 15 | 9.2195 | SEEG |
| Left Fusiform | 16 | 2 | SEEG |
| Right Inferior Parietal | 16 | 1 | SEEG |
| Right Inferior Parietal | 16 | 1.4142 | SEEG |
| Right Inferior Parietal | 16 | 1.7321 | SEEG |
| Right Inferior Parietal | 16 | 4.899 | SEEG |
| Right Postcentral | 16 | 3.7417 | SEEG |
| Right Superior Parietal | 16 | 5.7446 | SEEG |
| Left Precentral | 17 | 3 | SEEG |
| Left Precentral | 17 | 5.3852 | SEEG |
| Right Lateral Orbitofrontal | 17 | 2.2361 | SEEG |
| Right Lateral Orbitofrontal | 17 | 3.1623 | SEEG |
| Right Lateral Orbitofrontal | 17 | 5.099 | SEEG |
| Right Medial Orbitofrontal | 17 | 1.4142 | SEEG |
| Right Medial Orbitofrontal | 17 | 3.6056 | SEEG |
| Right Medial Orbitofrontal | 17 | 4.5826 | SEEG |
| Right Precentral | 17 | 4.5826 | SEEG |
| Right Superior Frontal | 17 | 1 | SEEG |
| Right Superior Frontal | 17 | 1.4142 | SEEG |
| Right Superior Frontal | 17 | 3 | SEEG |
| Right Superior Frontal | 17 | 3.1623 | SEEG |
| Right Superior Frontal | 17 | 5 | SEEG |
| Left Fusiform | 18 | 1 | SEEG |
| Left Fusiform | 18 | 3.1623 | SEEG |
| Left Inferior Parietal | 18 | 2.2361 | SEEG |
| Left Middle Temporal | 18 | 2.2361 | SEEG |
| Right Inferior Temporal | 18 | 1.7321 | SEEG |
| Right Middle Temporal | 18 | 1.7321 | SEEG |
| Right Pars Orbitalis | 18 | 2 | SEEG |
| Right Precuneus | 18 | 3.6056 | SEEG |
| Right Superior Frontal | 18 | 1.4142 | SEEG |
| Right Superior Frontal | 18 | 2 | SEEG |
| Right Superior Frontal | 18 | 2.2361 | SEEG |
| Right Superior Frontal | 18 | 4.2426 | SEEG |
| Right Superior Parietal | 18 | 2.2361 | SEEG |
| Right Superior Temporal | 18 | 2.2361 | SEEG |
| Left Banks of the Superior Temporal Sulcus | 19 | 2 | SEEG |
| Left Fusiform | 19 | 0 | SEEG |
| Left Fusiform | 19 | 2.2361 | SEEG |
| Left Inferior Temporal | 19 | 1 | SEEG |
| Left Inferior Temporal | 19 | 2 | SEEG |
| Left Inferior Temporal | 19 | 4.3589 | SEEG |
| Left Inferior Temporal | 19 | 5.1962 | SEEG |
| Left Inferior Temporal | 19 | 7.4833 | SEEG |
| Left Lateral Orbitofrontal | 19 | 1.4142 | SEEG |
| Left Middle Temporal | 19 | 2.8284 | SEEG |
| Left Middle Temporal | 19 | 3.1623 | SEEG |
| Left Middle Temporal | 19 | 6.7082 | SEEG |
| Left Pars Orbitalis | 19 | 1 | SEEG |
| Left Pars Orbitalis | 19 | 4.1231 | SEEG |
| Left Superior Temporal | 19 | 1.4142 | SEEG |
| Left Entorhinal | 20 | 1 | SEEG |
| Left Fusiform | 20 | 0 | SEEG |
| Left Fusiform | 20 | 1 | SEEG |
| Left Fusiform | 20 | 2 | SEEG |
| Left Inferior Temporal | 20 | 1 | SEEG |
| Left Inferior Temporal | 20 | 1.4142 | SEEG |
| Left Inferior Temporal | 20 | 3 | SEEG |
| Left Lateral Orbitofrontal | 20 | 1 | SEEG |
| Left Middle Temporal | 20 | 0 | SEEG |
| Left Pars Triangularis | 20 | 2.4495 | SEEG |
| Left Pars Triangularis | 20 | 3 | SEEG |
| Left Superior Temporal | 20 | 0 | SEEG |
| Left Superior Temporal | 20 | 3.1623 | SEEG |
| Right Fusiform | 20 | 0 | SEEG |
| Right Inferior Temporal | 20 | 0 | SEEG |
| Right Inferior Temporal | 20 | 3.6056 | SEEG |
| Right Middle Temporal | 20 | 1.7321 | SEEG |
| Right Rostral Middle Frontal | 20 | 0 | SEEG |
| Right Rostral Middle Frontal | 20 | 1 | SEEG |
| Right Superior Frontal | 20 | 0 | SEEG |
| Right Superior Frontal | 20 | 1 | SEEG |
| Right Superior Frontal | 20 | 2 | SEEG |
| Right Superior Frontal | 20 | 2.8284 | SEEG |
| Right Superior Frontal | 20 | 3.1623 | SEEG |
| Right Superior Frontal | 20 | 3.4641 | SEEG |
| Left Fusiform | 21 | 1 | SEEG |
| Left Fusiform | 21 | 2.2361 | SEEG |
| Left Fusiform | 21 | 5 | SEEG |
| Left Fusiform | 21 | 5.3852 | SEEG |
| Left Inferior Temporal | 21 | 3 | SEEG |
| Left Inferior Temporal | 21 | 4.2426 | SEEG |
| Left Lateral Orbitofrontal | 21 | 1.4142 | SEEG |
| Left Middle Temporal | 21 | 1.7321 | SEEG |
| Left Middle Temporal | 21 | 4.5826 | SEEG |
| Left Pars Orbitalis | 21 | 1 | SEEG |
| Left Pars Orbitalis | 21 | 4.2426 | SEEG |
| Left Pars Triangularis | 21 | 4.1231 | SEEG |
| Right Inferior Temporal | 21 | 0 | SEEG |
| Right Inferior Temporal | 21 | 1 | SEEG |
| Right Inferior Temporal | 21 | 1.4142 | SEEG |
| Right Lateral Orbitofrontal | 21 | 3 | SEEG |
| Right Medial Orbitofrontal | 21 | 3.7417 | SEEG |
| Right Medial Orbitofrontal | 21 | 4 | SEEG |
| Right Middle Temporal | 21 | 2.2361 | SEEG |
| Right Rostral Middle Frontal | 21 | 6.1644 | SEEG |
| Right Superior Frontal | 21 | 1 | SEEG |
| Right Superior Frontal | 21 | 2 | SEEG |
| Right Superior Frontal | 21 | 2.2361 | SEEG |
| Right Superior Frontal | 21 | 3 | SEEG |
| Right Superior Frontal | 21 | 3.1623 | SEEG |
| Right Superior Frontal | 21 | 3.6056 | SEEG |
| Right Superior Frontal | 21 | 3.7417 | SEEG |
| Right Superior Frontal | 21 | 4.5826 | SEEG |
| Left Caudal Middle Frontal | 22 | 3.3166 | SEEG |
| Left Caudal Middle Frontal | 22 | 4 | SEEG |
| Left Caudal Middle Frontal | 22 | 4.4721 | SEEG |
| Left Caudal Middle Frontal | 22 | 5.831 | SEEG |
| Left Fusiform | 22 | 3.7417 | SEEG |
| Left Fusiform | 22 | 5.099 | SEEG |
| Left Fusiform | 22 | 5.7446 | SEEG |
| Left Fusiform | 22 | 6.9282 | SEEG |
| Left Fusiform | 22 | 8.124 | SEEG |
| Left Inferior Temporal | 22 | 4.1231 | SEEG |
| Left Inferior Temporal | 22 | 5.099 | SEEG |
| Left Inferior Temporal | 22 | 5.7446 | SEEG |
| Left Inferior Temporal | 22 | 5.9161 | SEEG |
| Left Lateral Orbitofrontal | 22 | 0 | SEEG |
| Left Lateral Orbitofrontal | 22 | 1 | SEEG |
| Left Lateral Orbitofrontal | 22 | 2.2361 | SEEG |
| Left Lateral Orbitofrontal | 22 | 6.1644 | SEEG |
| Left Middle Temporal | 22 | 2.2361 | SEEG |
| Left Pars Orbitalis | 22 | 5.9161 | SEEG |
| Left Pars Triangularis | 22 | 1 | SEEG |
| Left Pars Triangularis | 22 | 3 | SEEG |
| Left Precentral | 22 | 7.8102 | SEEG |
| Left Rostral Middle Frontal | 22 | 5.9161 | SEEG |
| Right Fusiform | 22 | 3.4641 | SEEG |
| Right Fusiform | 22 | 5.7446 | SEEG |
| Right Fusiform | 22 | 5.831 | SEEG |
| Right Fusiform | 22 | 7.874 | SEEG |
| Right Fusiform | 22 | 9.9499 | SEEG |
| Right Inferior Temporal | 22 | 7.8102 | SEEG |
| Right Inferior Temporal | 22 | 12.0416 | SEEG |
| Right Lateral Orbitofrontal | 22 | 2 | SEEG |
| Right Medial Orbitofrontal | 22 | 4 | SEEG |
| Right Middle Temporal | 22 | 2 | SEEG |
| Right Middle Temporal | 22 | 3 | SEEG |
| Right Middle Temporal | 22 | 3.6056 | SEEG |
| Right Middle Temporal | 22 | 4.2426 | SEEG |
| Right Middle Temporal | 22 | 5.7446 | SEEG |
| Right Middle Temporal | 22 | 6 | SEEG |
| Right Pars Opercularis | 22 | 1.4142 | SEEG |
| Right Pars Opercularis | 22 | 2 | SEEG |
| Right Pars Opercularis | 22 | 5.099 | SEEG |
| Right Pars Orbitalis | 22 | 1.4142 | SEEG |
| Right Pars Triangularis | 22 | 3.4641 | SEEG |
| Right Precuneus | 22 | 4.1231 | SEEG |
| Right Precuneus | 22 | 6.4031 | SEEG |
| Right Rostral Middle Frontal | 22 | 0 | SEEG |
| Right Rostral Middle Frontal | 22 | 2.2361 | SEEG |
| Right Rostral Middle Frontal | 22 | 2.8284 | SEEG |
| Right Rostral Middle Frontal | 22 | 4.4721 | SEEG |
| Right Superior Frontal | 22 | 1.4142 | SEEG |
| Right Superior Frontal | 22 | 2 | SEEG |
| Right Superior Frontal | 22 | 2.4495 | SEEG |
| Right Superior Frontal | 22 | 4.3589 | SEEG |
| Right Superior Frontal | 22 | 5.099 | SEEG |
| Right Superior Frontal | 22 | 5.7446 | SEEG |
| Right Superior Parietal | 22 | 1 | SEEG |
| Right Superior Parietal | 22 | 3 | SEEG |
| Left Fusiform | 23 | 3 | SEEG |
| Left Fusiform | 23 | 6.7082 | SEEG |
| Left Fusiform | 23 | 10.0499 | SEEG |
| Left Inferior Temporal | 23 | 6.1644 | SEEG |
| Left Inferior Temporal | 23 | 7.3485 | SEEG |
| Left Insula | 23 | 4.3589 | SEEG |
| Left Lateral Orbitofrontal | 23 | 0 | SEEG |
| Left Lateral Orbitofrontal | 23 | 1.4142 | SEEG |
| Left Lateral Orbitofrontal | 23 | 1.7321 | SEEG |
| Left Lateral Orbitofrontal | 23 | 2.2361 | SEEG |
| Left Lateral Orbitofrontal | 23 | 2.4495 | SEEG |
| Left Middle Temporal | 23 | 2.2361 | SEEG |
| Left Middle Temporal | 23 | 8.3066 | SEEG |
| Left Pars Triangularis | 23 | 2.4495 | SEEG |
| Left Precentral | 23 | 5.4772 | SEEG |
| Left Precentral | 23 | 7.6811 | SEEG |
| Left Rostral Middle Frontal | 23 | 2 | SEEG |
| Left Superior Temporal | 23 | 2.8284 | SEEG |
| Left Transverse Temporal | 23 | 9.1104 | SEEG |
| Right Caudal Middle Frontal | 23 | 2.2361 | SEEG |
| Right Fusiform | 23 | 3.1623 | SEEG |
| Right Fusiform | 23 | 5.099 | SEEG |
| Right Inferior Temporal | 23 | 1.4142 | SEEG |
| Right Inferior Temporal | 23 | 2 | SEEG |
| Right Inferior Temporal | 23 | 2.8284 | SEEG |
| Right Inferior Temporal | 23 | 3.1623 | SEEG |
| Right Lateral Orbitofrontal | 23 | 1.4142 | SEEG |
| Right Lateral Orbitofrontal | 23 | 2 | SEEG |
| Right Lateral Orbitofrontal | 23 | 2.8284 | SEEG |
| Right Lateral Orbitofrontal | 23 | 3 | SEEG |
| Right Middle Temporal | 23 | 0 | SEEG |
| Right Middle Temporal | 23 | 1.4142 | SEEG |
| Right Precentral | 23 | 4.1231 | SEEG |
| Right Rostral Middle Frontal | 23 | 0 | SEEG |
| Right Rostral Middle Frontal | 23 | 1 | SEEG |
| Right Rostral Middle Frontal | 23 | 2.2361 | SEEG |
| Right Rostral Middle Frontal | 23 | 2.4495 | SEEG |
| Right Superior Frontal | 23 | 1 | SEEG |
| Right Superior Frontal | 23 | 2 | SEEG |
| Right Superior Frontal | 23 | 2.4495 | SEEG |
| Right Superior Frontal | 23 | 3 | SEEG |
| Right Superior Frontal | 23 | 3.7417 | SEEG |
| Right Superior Temporal | 23 | 2 | SEEG |
| Left Fusiform | 24 | 5 | SEEG |
| Left Lateral Orbitofrontal | 24 | 2.8284 | SEEG |
| Left Lateral Orbitofrontal | 24 | 3 | SEEG |
| Left Lateral Orbitofrontal | 24 | 4.5826 | SEEG |
| Left Middle Temporal | 24 | 1 | SEEG |
| Left Middle Temporal | 24 | 4.5826 | SEEG |
| Left Pars Triangularis | 24 | 5 | SEEG |
| Right Lateral Orbitofrontal | 24 | 2.2361 | SEEG |
| Right Lateral Orbitofrontal | 24 | 2.8284 | SEEG |
| Right Lateral Orbitofrontal | 24 | 3 | SEEG |
| Right Lateral Orbitofrontal | 24 | 5 | SEEG |
| Right Pars Triangularis | 24 | 0 | SEEG |
| Right Pars Triangularis | 24 | 3 | SEEG |
| Right Superior Frontal | 24 | 0 | SEEG |
| Right Superior Frontal | 24 | 1.4142 | SEEG |
| Right Superior Frontal | 24 | 3.3166 | SEEG |
| Right Superior Frontal | 24 | 5 | SEEG |
| Right Superior Temporal | 24 | 2.2361 | SEEG |
| Right Fusiform | 25 | 6 | SEEG |
| Right Fusiform | 25 | 7.2111 | SEEG |
| Right Fusiform | 25 | 10.0499 | SEEG |
| Right Fusiform | 25 | 10.6301 | SEEG |
| Right Inferior Temporal | 25 | 3.4641 | SEEG |
| Right Inferior Temporal | 25 | 9.4868 | SEEG |
| Right Lateral Orbitofrontal | 25 | 1.4142 | SEEG |
| Right Lateral Orbitofrontal | 25 | 2.2361 | SEEG |
| Right Lateral Orbitofrontal | 25 | 3 | SEEG |
| Right Lateral Orbitofrontal | 25 | 4.4721 | SEEG |
| Right Middle Temporal | 25 | 0 | SEEG |
| Right Middle Temporal | 25 | 1.4142 | SEEG |
| Right Middle Temporal | 25 | 2.2361 | SEEG |
| Right Middle Temporal | 25 | 2.4495 | SEEG |
| Right Middle Temporal | 25 | 3 | SEEG |
| Right Middle Temporal | 25 | 3.6056 | SEEG |
| Right Middle Temporal | 25 | 7.2801 | SEEG |
| Right Middle Temporal | 25 | 10.9545 | SEEG |
| Right Superior Temporal | 25 | 2.4495 | SEEG |
| Right Superior Temporal | 25 | 7 | SEEG |
| Left Banks of the Superior Temporal Sulcus | 26 | 2 | SEEG |
| Left Banks of the Superior Temporal Sulcus | 26 | 3.6056 | SEEG |
| Left Caudal Middle Frontal | 26 | 2.2361 | SEEG |
| Left Caudal Middle Frontal | 26 | 3.7417 | SEEG |
| Left Fusiform | 26 | 3.7417 | SEEG |
| Left Fusiform | 26 | 4 | SEEG |
| Left Fusiform | 26 | 9.0554 | SEEG |
| Left Inferior Temporal | 26 | 5.099 | SEEG |
| Left Middle Temporal | 26 | 2.4495 | SEEG |
| Left Middle Temporal | 26 | 3.6056 | SEEG |
| Left Pars Orbitalis | 26 | 0 | SEEG |
| Left Pars Orbitalis | 26 | 1 | SEEG |
| Left Precentral | 26 | 5.4772 | SEEG |
| Left Superior Temporal | 26 | 4 | SEEG |
| Right Caudal Middle Frontal | 26 | 1 | SEEG |
| Right Caudal Middle Frontal | 26 | 3 | SEEG |
| Right Caudal Middle Frontal | 26 | 3.1623 | SEEG |
| Right Caudal Middle Frontal | 26 | 4.3589 | SEEG |
| Right Fusiform | 26 | 1 | SEEG |
| Right Fusiform | 26 | 3.1623 | SEEG |
| Right Inferior Temporal | 26 | 1.7321 | SEEG |
| Right Inferior Temporal | 26 | 3.7417 | SEEG |
| Right Inferior Temporal | 26 | 4.1231 | SEEG |
| Right Inferior Temporal | 26 | 5.9161 | SEEG |
| Right Insula | 26 | 3 | SEEG |
| Right Insula | 26 | 3.4641 | SEEG |
| Right Middle Temporal | 26 | 4.5826 | SEEG |
| Right Precentral | 26 | 2.4495 | SEEG |
| Right Precentral | 26 | 2.8284 | SEEG |
| Right Precentral | 26 | 3.7417 | SEEG |
| Right Precentral | 26 | 7.5498 | SEEG |
| Right Precuneus | 26 | 1.4142 | SEEG |
| Right Precuneus | 26 | 4.5826 | SEEG |
| Right Rostral Middle Frontal | 26 | 0 | SEEG |
| Right Rostral Middle Frontal | 26 | 2.2361 | SEEG |
| Right Superior Frontal | 26 | 1 | SEEG |
| Right Superior Frontal | 26 | 1.4142 | SEEG |
| Right Superior Parietal | 26 | 2.4495 | SEEG |
| Right Superior Parietal | 26 | 3.1623 | SEEG |
| Right Superior Parietal | 26 | 5.3852 | SEEG |

**LR**

| **Location** | **Subject** | **VMD** | **Type** |
| --- | --- | --- | --- |
| Right Middle Temporal | 9 | 2.4495 | SEEG |
| Right Superior Temporal | 9 | 1 | SEEG |
| Left Inferior Parietal | 7 | 0 | Grid |
| Left Inferior Temporal | 7 | 0 | Grid |
| Left Middle Temporal | 7 | 0 | Grid |
| Left Pars Opercularis | 7 | 0 | Grid |
| Left Pars Orbitalis | 7 | 0 | Grid |
| Left Pars Triangularis | 7 | 0 | Grid |
| Left Postcentral | 7 | 0 | Grid |
| Left Precentral | 7 | 0 | Grid |
| Left Rostral Middle Frontal | 7 | 0 | Grid |
| Left Superior Temporal | 7 | 0 | Grid |
| Right Inferior Parietal | 6 | 0 | Grid |
| Right Lateral Orbitofrontal | 6 | 0 | Grid |
| Right Pars Opercularis | 6 | 0 | Grid |
| Right Pars Triangularis | 6 | 0 | Grid |
| Right Postcentral | 6 | 0 | Grid |
| Right Precentral | 6 | 0 | Grid |
| Right Superior Temporal | 6 | 0 | Grid |
| Right Supramarginal | 6 | 0 | Grid |
| Right Transverse Temporal | 6 | 0 | Grid |
| Right Fusiform | 6 | 0 | Strip |
| Right Parahippocampal | 6 | 0 | Strip |
| Left Fusiform | 24 | 5 | SEEG |
| Left Lateral Orbitofrontal | 24 | 2.8284 | SEEG |
| Left Lateral Orbitofrontal | 24 | 3 | SEEG |
| Left Lateral Orbitofrontal | 24 | 4.5826 | SEEG |
| Left Middle Temporal | 24 | 1 | SEEG |
| Left Middle Temporal | 24 | 4.5826 | SEEG |
| Left Pars Triangularis | 24 | 5 | SEEG |
| Right Lateral Orbitofrontal | 24 | 2.2361 | SEEG |
| Right Lateral Orbitofrontal | 24 | 2.8284 | SEEG |
| Right Lateral Orbitofrontal | 24 | 3 | SEEG |
| Right Lateral Orbitofrontal | 24 | 5 | SEEG |
| Right Pars Triangularis | 24 | 3 | SEEG |
| Right Superior Frontal | 24 | 1.4142 | SEEG |
| Right Superior Frontal | 24 | 3.3166 | SEEG |
| Right Superior Frontal | 24 | 5 | SEEG |
| Right Superior Temporal | 24 | 2.2361 | SEEG |
| Left Precentral | 17 | 3 | SEEG |
| Left Precentral | 17 | 5.3852 | SEEG |
| Right Lateral Orbitofrontal | 17 | 2.2361 | SEEG |
| Right Lateral Orbitofrontal | 17 | 3.1623 | SEEG |
| Right Lateral Orbitofrontal | 17 | 5.099 | SEEG |
| Right Precentral | 17 | 4.5826 | SEEG |
| Right Superior Frontal | 17 | 1 | SEEG |
| Right Superior Frontal | 17 | 1.4142 | SEEG |
| Right Superior Frontal | 17 | 3 | SEEG |
| Right Superior Frontal | 17 | 3.1623 | SEEG |
| Right Superior Frontal | 17 | 5 | SEEG |
| Right Transverse Temporal | 4 | 7.874 | Depth |
| Right Inferior Parietal | 4 | 0 | Grid |
| Right Lateral Occipital | 4 | 0 | Grid |
| Right Middle Temporal | 4 | 0 | Grid |
| Right Pars Opercularis | 4 | 0 | Grid |
| Right Pars Triangularis | 4 | 0 | Grid |
| Right Postcentral | 4 | 0 | Grid |
| Right Precentral | 4 | 0 | Grid |
| Right Rostral Middle Frontal | 4 | 0 | Grid |
| Right Superior Parietal | 4 | 0 | Grid |
| Right Superior Temporal | 4 | 0 | Grid |
| Right Entorhinal | 4 | 0 | Strip |
| Right Fusiform | 4 | 0 | Strip |
| Right Parahippocampal | 4 | 0 | Strip |
| Left Fusiform | 13 | 2.2361 | SEEG |
| Left Fusiform | 13 | 3 | SEEG |
| Left Fusiform | 13 | 3.1623 | SEEG |
| Left Fusiform | 13 | 3.7417 | SEEG |
| Left Inferior Temporal | 13 | 3 | SEEG |
| Left Inferior Temporal | 13 | 5.099 | SEEG |
| Left Insula | 13 | 5.7446 | SEEG |
| Left Insula | 13 | 6 | SEEG |
| Left Middle Temporal | 13 | 5.099 | SEEG |
| Left Middle Temporal | 13 | 5.3852 | SEEG |
| Left Rostral Middle Frontal | 13 | 2 | SEEG |
| Left Rostral Middle Frontal | 13 | 3 | SEEG |
| Left Rostral Middle Frontal | 13 | 4.1231 | SEEG |
| Left Superior Temporal | 13 | 1.4142 | SEEG |
| Left Superior Temporal | 13 | 2.2361 | SEEG |
| Right Fusiform | 13 | 2.2361 | SEEG |
| Right Inferior Temporal | 13 | 6.1644 | SEEG |
| Right Inferior Temporal | 13 | 8.0623 | SEEG |
| Right Middle Temporal | 13 | 4.3589 | SEEG |
| Right Middle Temporal | 13 | 6.0828 | SEEG |
| Right Pars Triangularis | 13 | 4.5826 | SEEG |
| Right Rostral Middle Frontal | 13 | 4.4721 | SEEG |
| Left Fusiform | 23 | 6.7082 | SEEG |
| Left Fusiform | 23 | 10.0499 | SEEG |
| Left Inferior Temporal | 23 | 6.1644 | SEEG |
| Left Inferior Temporal | 23 | 7.3485 | SEEG |
| Left Insula | 23 | 4.3589 | SEEG |
| Left Lateral Orbitofrontal | 23 | 0 | SEEG |
| Left Lateral Orbitofrontal | 23 | 1.4142 | SEEG |
| Left Lateral Orbitofrontal | 23 | 1.7321 | SEEG |
| Left Lateral Orbitofrontal | 23 | 2.2361 | SEEG |
| Left Lateral Orbitofrontal | 23 | 2.4495 | SEEG |
| Left Pars Triangularis | 23 | 2.4495 | SEEG |
| Left Precentral | 23 | 5.4772 | SEEG |
| Left Rostral Middle Frontal | 23 | 2 | SEEG |
| Left Superior Temporal | 23 | 2.8284 | SEEG |
| Left Transverse Temporal | 23 | 9.1104 | SEEG |
| Right Fusiform | 23 | 5.099 | SEEG |
| Right Inferior Temporal | 23 | 1.4142 | SEEG |
| Right Inferior Temporal | 23 | 2 | SEEG |
| Right Inferior Temporal | 23 | 2.8284 | SEEG |
| Right Inferior Temporal | 23 | 3.1623 | SEEG |
| Right Lateral Orbitofrontal | 23 | 1.4142 | SEEG |
| Right Lateral Orbitofrontal | 23 | 2 | SEEG |
| Right Lateral Orbitofrontal | 23 | 2.8284 | SEEG |
| Right Lateral Orbitofrontal | 23 | 3 | SEEG |
| Right Middle Temporal | 23 | 0 | SEEG |
| Right Middle Temporal | 23 | 1.4142 | SEEG |
| Right Precentral | 23 | 4.1231 | SEEG |
| Right Rostral Middle Frontal | 23 | 0 | SEEG |
| Right Rostral Middle Frontal | 23 | 1 | SEEG |
| Right Rostral Middle Frontal | 23 | 2.2361 | SEEG |
| Right Rostral Middle Frontal | 23 | 2.4495 | SEEG |
| Right Superior Frontal | 23 | 1 | SEEG |
| Right Superior Frontal | 23 | 2 | SEEG |
| Right Superior Frontal | 23 | 2.4495 | SEEG |
| Right Superior Frontal | 23 | 3 | SEEG |
| Right Superior Frontal | 23 | 3.7417 | SEEG |
| Right Superior Temporal | 23 | 2 | SEEG |
| Left Fusiform | 12 | 3.4641 | SEEG |
| Left Fusiform | 12 | 9.6437 | SEEG |
| Left Inferior Parietal | 12 | 8.6023 | SEEG |
| Left Inferior Parietal | 12 | 10.198 | SEEG |
| Left Inferior Parietal | 12 | 14.1774 | SEEG |
| Left Inferior Parietal | 12 | 15.3623 | SEEG |
| Right Superior Temporal | 12 | 4.1231 | SEEG |
| Right Superior Temporal | 12 | 8.9443 | SEEG |
| Right Superior Temporal | 12 | 10.2956 | SEEG |
| Right Fusiform | 25 | 6 | SEEG |
| Right Fusiform | 25 | 7.2111 | SEEG |
| Right Fusiform | 25 | 10.0499 | SEEG |
| Right Fusiform | 25 | 10.6301 | SEEG |
| Right Inferior Temporal | 25 | 3.4641 | SEEG |
| Right Inferior Temporal | 25 | 9.4868 | SEEG |
| Right Lateral Orbitofrontal | 25 | 1.4142 | SEEG |
| Right Lateral Orbitofrontal | 25 | 2.2361 | SEEG |
| Right Lateral Orbitofrontal | 25 | 3 | SEEG |
| Right Lateral Orbitofrontal | 25 | 4.4721 | SEEG |
| Right Middle Temporal | 25 | 1.4142 | SEEG |
| Right Middle Temporal | 25 | 2.2361 | SEEG |
| Right Middle Temporal | 25 | 2.4495 | SEEG |
| Right Middle Temporal | 25 | 3.6056 | SEEG |
| Right Middle Temporal | 25 | 7.2801 | SEEG |
| Right Middle Temporal | 25 | 10.9545 | SEEG |
| Right Superior Temporal | 25 | 2.4495 | SEEG |
| Right Superior Temporal | 25 | 7 | SEEG |
| Left Caudal Middle Frontal | 10 | 3.1623 | SEEG |
| Left Caudal Middle Frontal | 10 | 4.1231 | SEEG |
| Left Caudal Middle Frontal | 10 | 6.7082 | SEEG |
| Left Fusiform | 10 | 7.3485 | SEEG |
| Left Inferior Parietal | 10 | 4.2426 | SEEG |
| Left Lateral Orbitofrontal | 10 | 4.3589 | SEEG |
| Left Middle Temporal | 10 | 1.4142 | SEEG |
| Left Middle Temporal | 10 | 3.6056 | SEEG |
| Left Middle Temporal | 10 | 4.5826 | SEEG |
| Left Middle Temporal | 10 | 5 | SEEG |
| Left Middle Temporal | 10 | 5.3852 | SEEG |
| Left Pars Orbitalis | 10 | 0 | SEEG |
| Left Fusiform | 20 | 0 | SEEG |
| Left Fusiform | 20 | 1 | SEEG |
| Left Fusiform | 20 | 2 | SEEG |
| Left Inferior Temporal | 20 | 1 | SEEG |
| Left Inferior Temporal | 20 | 1.4142 | SEEG |
| Left Inferior Temporal | 20 | 3 | SEEG |
| Left Lateral Orbitofrontal | 20 | 1 | SEEG |
| Left Pars Triangularis | 20 | 2.4495 | SEEG |
| Left Superior Temporal | 20 | 0 | SEEG |
| Left Superior Temporal | 20 | 3.1623 | SEEG |
| Right Fusiform | 20 | 0 | SEEG |
| Right Inferior Temporal | 20 | 0 | SEEG |
| Right Inferior Temporal | 20 | 3.6056 | SEEG |
| Right Middle Temporal | 20 | 1.7321 | SEEG |
| Right Rostral Middle Frontal | 20 | 0 | SEEG |
| Right Superior Frontal | 20 | 1 | SEEG |
| Right Superior Frontal | 20 | 2 | SEEG |
| Right Superior Frontal | 20 | 2.8284 | SEEG |
| Right Superior Frontal | 20 | 3.1623 | SEEG |
| Right Superior Frontal | 20 | 3.4641 | SEEG |
| Left Caudal Middle Frontal | 11 | 1.4142 | SEEG |
| Left Caudal Middle Frontal | 11 | 2.2361 | SEEG |
| Left Caudal Middle Frontal | 11 | 2.4495 | SEEG |
| Left Caudal Middle Frontal | 11 | 3.1623 | SEEG |
| Left Caudal Middle Frontal | 11 | 3.3166 | SEEG |
| Left Caudal Middle Frontal | 11 | 4.1231 | SEEG |
| Left Fusiform | 11 | 0 | SEEG |
| Left Inferior Parietal | 11 | 1.4142 | SEEG |
| Left Inferior Parietal | 11 | 2.2361 | SEEG |
| Left Inferior Parietal | 11 | 3 | SEEG |
| Left Inferior Temporal | 11 | 1 | SEEG |
| Left Inferior Temporal | 11 | 2 | SEEG |
| Left Insula | 11 | 1 | SEEG |
| Left Insula | 11 | 1.4142 | SEEG |
| Left Insula | 11 | 1.7321 | SEEG |
| Left Insula | 11 | 2 | SEEG |
| Left Insula | 11 | 4.899 | SEEG |
| Left Precentral | 11 | 5 | SEEG |
| Left Precentral | 11 | 8.1854 | SEEG |
| Left Precentral | 11 | 9.2195 | SEEG |
| Left Rostral Middle Frontal | 11 | 3.1623 | SEEG |
| Right Caudal Middle Frontal | 11 | 0 | SEEG |
| Right Caudal Middle Frontal | 11 | 1 | SEEG |
| Right Caudal Middle Frontal | 11 | 2.4495 | SEEG |
| Right Caudal Middle Frontal | 11 | 3.1623 | SEEG |
| Right Inferior Parietal | 11 | 0 | SEEG |
| Right Inferior Parietal | 11 | 1 | SEEG |
| Right Insula | 11 | 1.4142 | SEEG |
| Right Insula | 11 | 6.0828 | SEEG |
| Right Middle Temporal | 11 | 2.4495 | SEEG |
| Right Precentral | 11 | 1 | SEEG |
| Right Precentral | 11 | 3 | SEEG |
| Right Precentral | 11 | 4.2426 | SEEG |
| Right Precentral | 11 | 4.3589 | SEEG |
| Right Superior Parietal | 11 | 4.2426 | SEEG |
| Right Superior Temporal | 11 | 0 | SEEG |
| Left Inferior Temporal | 14 | 1 | SEEG |
| Left Insula | 14 | 2 | SEEG |
| Left Insula | 14 | 2.4495 | SEEG |
| Left Lateral Orbitofrontal | 14 | 2.2361 | SEEG |
| Left Middle Temporal | 14 | 3.7417 | SEEG |
| Left Pars Triangularis | 14 | 2.2361 | SEEG |
| Left Superior Temporal | 14 | 2.2361 | SEEG |
| Right Inferior Temporal | 14 | 1 | SEEG |
| Right Middle Temporal | 14 | 3 | SEEG |
| Right Middle Temporal | 14 | 4.1231 | SEEG |
| Right Pars Opercularis | 14 | 3.3166 | SEEG |
| Right Pars Triangularis | 14 | 1.7321 | SEEG |
| Right Pars Triangularis | 14 | 3 | SEEG |
| Right Rostral Middle Frontal | 14 | 0 | SEEG |
| Right Rostral Middle Frontal | 14 | 1 | SEEG |
| Right Rostral Middle Frontal | 14 | 1.7321 | SEEG |
| Right Superior Frontal | 14 | 1.4142 | SEEG |
| Right Superior Frontal | 14 | 2 | SEEG |
| Right Superior Temporal | 14 | 0 | SEEG |
| Right Superior Temporal | 14 | 1.7321 | SEEG |
| Right Superior Temporal | 14 | 2.8284 | SEEG |
| Right Caudal Middle Frontal | 1 | 0 | Grid |
| Right Inferior Parietal | 1 | 0 | Grid |
| Right Inferior Temporal | 1 | 0 | Grid |
| Right Insula | 1 | 0 | Grid |
| Right Middle Temporal | 1 | 0 | Grid |
| Right Pars Opercularis | 1 | 0 | Grid |
| Right Postcentral | 1 | 0 | Grid |
| Right Precentral | 1 | 0 | Grid |
| Right Superior Temporal | 1 | 0 | Grid |
| Right Supramarginal | 1 | 0 | Grid |
| Right Fusiform | 1 | 0 | Strip |
| Right Parahippocampal | 1 | 0 | Strip |
| Right Rostral Middle Frontal | 1 | 0 | Strip |
| Right Inferior Parietal | 1 | 1 | SEEG |
| Right Inferior Parietal | 1 | 3.6056 | SEEG |
| Right Inferior Parietal | 1 | 4.1231 | SEEG |
| Right Inferior Parietal | 1 | 5.4772 | SEEG |
| Right Inferior Parietal | 1 | 6.4031 | SEEG |
| Right Inferior Parietal | 1 | 8.4853 | SEEG |
| Right Inferior Temporal | 1 | 1 | SEEG |
| Right Inferior Temporal | 1 | 2.8284 | SEEG |
| Right Inferior Temporal | 1 | 8.6023 | SEEG |
| Right Lateral Orbitofrontal | 1 | 1 | SEEG |
| Right Lateral Orbitofrontal | 1 | 4 | SEEG |
| Right Middle Temporal | 1 | 1.4142 | SEEG |
| Right Middle Temporal | 1 | 3.7417 | SEEG |
| Right Pars Triangularis | 1 | 3.1623 | SEEG |
| Right Precuneus | 1 | 6.4031 | SEEG |
| Right Superior Parietal | 1 | 6.7082 | SEEG |
| Right Superior Parietal | 1 | 7.0711 | SEEG |
| Right Inferior Temporal | 3 | 8.544 | Depth |
| Right Middle Temporal | 3 | 12.6886 | Depth |
| Right Transverse Temporal | 3 | 12.083 | Depth |
| Right Inferior Parietal | 3 | 0 | Grid |
| Right Postcentral | 3 | 0 | Grid |
| Right Precentral | 3 | 0 | Grid |
| Right Superior Parietal | 3 | 0 | Grid |
| Right Supramarginal | 3 | 0 | Grid |
| Right Caudal Anterior Cingulate | 3 | 0 | Strip |
| Right Caudal Middle Frontal | 3 | 0 | Strip |
| Right Entorhinal | 3 | 0 | Strip |
| Right Inferior Temporal | 3 | 0 | Strip |
| Right Insula | 3 | 0 | Strip |
| Right Lateral Occipital | 3 | 0 | Strip |
| Right Middle Temporal | 3 | 0 | Strip |
| Right Superior Frontal | 3 | 0 | Strip |
| Right Superior Temporal | 3 | 0 | Strip |
| Right Temporal Pole | 3 | 0 | Strip |
| Left Fusiform | 21 | 2.2361 | SEEG |
| Left Fusiform | 21 | 5 | SEEG |
| Left Fusiform | 21 | 5.3852 | SEEG |
| Left Inferior Temporal | 21 | 3 | SEEG |
| Left Inferior Temporal | 21 | 4.2426 | SEEG |
| Left Lateral Orbitofrontal | 21 | 1.4142 | SEEG |
| Left Middle Temporal | 21 | 4.5826 | SEEG |
| Left Pars Orbitalis | 21 | 1 | SEEG |
| Left Pars Orbitalis | 21 | 4.2426 | SEEG |
| Right Caudal Anterior Cingulate | 21 | 5.099 | SEEG |
| Right Inferior Temporal | 21 | 0 | SEEG |
| Right Inferior Temporal | 21 | 1 | SEEG |
| Right Inferior Temporal | 21 | 1.4142 | SEEG |
| Right Lateral Orbitofrontal | 21 | 3 | SEEG |
| Right Middle Temporal | 21 | 2.2361 | SEEG |
| Right Rostral Middle Frontal | 21 | 6.1644 | SEEG |
| Right Superior Frontal | 21 | 1 | SEEG |
| Right Superior Frontal | 21 | 2 | SEEG |
| Right Superior Frontal | 21 | 2.2361 | SEEG |
| Right Superior Frontal | 21 | 3 | SEEG |
| Right Superior Frontal | 21 | 3.1623 | SEEG |
| Right Superior Frontal | 21 | 3.6056 | SEEG |
| Right Superior Frontal | 21 | 3.7417 | SEEG |
| Right Superior Frontal | 21 | 4.5826 | SEEG |
| Left Caudal Middle Frontal | 22 | 4 | SEEG |
| Left Caudal Middle Frontal | 22 | 4.4721 | SEEG |
| Left Caudal Middle Frontal | 22 | 5.831 | SEEG |
| Left Fusiform | 22 | 3.7417 | SEEG |
| Left Fusiform | 22 | 5.099 | SEEG |
| Left Fusiform | 22 | 5.7446 | SEEG |
| Left Fusiform | 22 | 6.9282 | SEEG |
| Left Fusiform | 22 | 8.124 | SEEG |
| Left Inferior Temporal | 22 | 4.1231 | SEEG |
| Left Inferior Temporal | 22 | 5.7446 | SEEG |
| Left Inferior Temporal | 22 | 5.9161 | SEEG |
| Left Lateral Orbitofrontal | 22 | 0 | SEEG |
| Left Lateral Orbitofrontal | 22 | 1 | SEEG |
| Left Lateral Orbitofrontal | 22 | 2.2361 | SEEG |
| Left Lateral Orbitofrontal | 22 | 6.1644 | SEEG |
| Left Middle Temporal | 22 | 2.2361 | SEEG |
| Left Pars Orbitalis | 22 | 5.9161 | SEEG |
| Left Pars Triangularis | 22 | 1 | SEEG |
| Left Pars Triangularis | 22 | 3 | SEEG |
| Left Precentral | 22 | 7.8102 | SEEG |
| Left Rostral Middle Frontal | 22 | 5.9161 | SEEG |
| Right Caudal Anterior Cingulate | 22 | 5 | SEEG |
| Right Fusiform | 22 | 3.4641 | SEEG |
| Right Fusiform | 22 | 5.7446 | SEEG |
| Right Fusiform | 22 | 5.831 | SEEG |
| Right Fusiform | 22 | 7.874 | SEEG |
| Right Fusiform | 22 | 9.9499 | SEEG |
| Right Inferior Temporal | 22 | 7.8102 | SEEG |
| Right Inferior Temporal | 22 | 12.0416 | SEEG |
| Right Lateral Orbitofrontal | 22 | 2 | SEEG |
| Right Middle Temporal | 22 | 2 | SEEG |
| Right Middle Temporal | 22 | 3 | SEEG |
| Right Middle Temporal | 22 | 3.6056 | SEEG |
| Right Middle Temporal | 22 | 4.2426 | SEEG |
| Right Middle Temporal | 22 | 5.7446 | SEEG |
| Right Middle Temporal | 22 | 6 | SEEG |
| Right Pars Opercularis | 22 | 1.4142 | SEEG |
| Right Pars Opercularis | 22 | 2 | SEEG |
| Right Pars Triangularis | 22 | 3.4641 | SEEG |
| Right Precuneus | 22 | 4.1231 | SEEG |
| Right Rostral Middle Frontal | 22 | 0 | SEEG |
| Right Rostral Middle Frontal | 22 | 2.8284 | SEEG |
| Right Rostral Middle Frontal | 22 | 4.4721 | SEEG |
| Right Superior Frontal | 22 | 1.4142 | SEEG |
| Right Superior Frontal | 22 | 2 | SEEG |
| Right Superior Frontal | 22 | 2.4495 | SEEG |
| Right Superior Frontal | 22 | 4.3589 | SEEG |
| Right Superior Frontal | 22 | 5.099 | SEEG |
| Right Superior Frontal | 22 | 5.7446 | SEEG |
| Right Superior Parietal | 22 | 1 | SEEG |
| Right Superior Parietal | 22 | 3 | SEEG |
| Right Caudal Middle Frontal | 5 | 0 | Grid |
| Right Superior Frontal | 5 | 0 | Grid |
| Right Fusiform | 5 | 0 | Strip |
| Right Inferior Temporal | 5 | 0 | Strip |
| Right Precuneus | 5 | 0 | Strip |
| Left Caudal Middle Frontal | 15 | 4.4721 | SEEG |
| Left Fusiform | 15 | 3.4641 | SEEG |
| Left Inferior Temporal | 15 | 1.4142 | SEEG |
| Left Inferior Temporal | 15 | 3 | SEEG |
| Left Inferior Temporal | 15 | 4.2426 | SEEG |
| Left Inferior Temporal | 15 | 4.899 | SEEG |
| Left Insula | 15 | 1 | SEEG |
| Left Middle Temporal | 15 | 3.7417 | SEEG |
| Left Middle Temporal | 15 | 4.5826 | SEEG |
| Left Pars Opercularis | 15 | 5.3852 | SEEG |
| Left Precentral | 15 | 2.2361 | SEEG |
| Left Rostral Middle Frontal | 15 | 2.8284 | SEEG |
| Left Rostral Middle Frontal | 15 | 3 | SEEG |
| Left Rostral Middle Frontal | 15 | 4 | SEEG |
| Right Caudal Anterior Cingulate | 15 | 3.1623 | SEEG |
| Right Fusiform | 15 | 3.1623 | SEEG |
| Right Inferior Parietal | 15 | 0 | SEEG |
| Right Inferior Parietal | 15 | 2.4495 | SEEG |
| Right Inferior Parietal | 15 | 5.6569 | SEEG |
| Right Inferior Temporal | 15 | 2.4495 | SEEG |
| Right Inferior Temporal | 15 | 3 | SEEG |
| Right Inferior Temporal | 15 | 4.1231 | SEEG |
| Right Insula | 15 | 1.4142 | SEEG |
| Right Insula | 15 | 3 | SEEG |
| Right Lateral Occipital | 15 | 2.2361 | SEEG |
| Right Lateral Orbitofrontal | 15 | 1.7321 | SEEG |
| Right Pars Triangularis | 15 | 2.2361 | SEEG |
| Right Precuneus | 15 | 2.2361 | SEEG |
| Right Precuneus | 15 | 4.5826 | SEEG |
| Right Precuneus | 15 | 9.5394 | SEEG |
| Right Rostral Middle Frontal | 15 | 1.4142 | SEEG |
| Right Rostral Middle Frontal | 15 | 5.3852 | SEEG |
| Right Rostral Middle Frontal | 15 | 6.1644 | SEEG |
| Right Superior Frontal | 15 | 2 | SEEG |
| Right Superior Frontal | 15 | 2.8284 | SEEG |
| Right Superior Frontal | 15 | 3.1623 | SEEG |
| Right Superior Frontal | 15 | 3.6056 | SEEG |
| Right Superior Frontal | 15 | 4.1231 | SEEG |
| Right Superior Parietal | 15 | 9.2195 | SEEG |
| Left Fusiform | 16 | 2 | SEEG |
| Right Inferior Parietal | 16 | 1 | SEEG |
| Right Inferior Parietal | 16 | 1.4142 | SEEG |
| Right Inferior Parietal | 16 | 1.7321 | SEEG |
| Right Inferior Parietal | 16 | 4.899 | SEEG |
| Right Postcentral | 16 | 3.7417 | SEEG |
| Right Superior Parietal | 16 | 5.7446 | SEEG |
| Left Fusiform | 2 | 2.2361 | SEEG |
| Left Fusiform | 2 | 4.1231 | SEEG |
| Left Fusiform | 2 | 4.899 | SEEG |
| Left Fusiform | 2 | 6.1644 | SEEG |
| Left Inferior Parietal | 2 | 1 | SEEG |
| Left Inferior Parietal | 2 | 2.4495 | SEEG |
| Left Inferior Parietal | 2 | 3.1623 | SEEG |
| Left Inferior Temporal | 2 | 2.2361 | SEEG |
| Left Inferior Temporal | 2 | 3.7417 | SEEG |
| Left Inferior Temporal | 2 | 4.1231 | SEEG |
| Left Lateral Orbitofrontal | 2 | 1 | SEEG |
| Left Pars Orbitalis | 2 | 2.2361 | SEEG |
| Left Pars Orbitalis | 2 | 2.8284 | SEEG |
| Left Pars Triangularis | 2 | 1.4142 | SEEG |
| Left Pars Triangularis | 2 | 2.8284 | SEEG |
| Left Pars Triangularis | 2 | 3 | SEEG |
| Left Postcentral | 2 | 11.5758 | SEEG |
| Left Precentral | 2 | 6.4031 | SEEG |
| Left Rostral Middle Frontal | 2 | 0 | SEEG |
| Left Rostral Middle Frontal | 2 | 1 | SEEG |
| Left Rostral Middle Frontal | 2 | 1.4142 | SEEG |
| Left Superior Temporal | 2 | 8.1854 | SEEG |
| Left Supramarginal | 2 | 4.2426 | SEEG |
| Left Insula | 2 | 0 | Grid |
| Left Pars Opercularis | 2 | 0 | Grid |
| Left Pars Triangularis | 2 | 0 | Grid |
| Left Postcentral | 2 | 0 | Grid |
| Left Rostral Middle Frontal | 2 | 0 | Grid |
| Left Superior Temporal | 2 | 0 | Grid |
| Left Supramarginal | 2 | 0 | Grid |
| Left Fusiform | 2 | 0 | Strip |
| Left Inferior Temporal | 2 | 0 | Strip |
| Left Lateral Orbitofrontal | 2 | 0 | Strip |
| Left Fusiform | 19 | 0 | SEEG |
| Left Fusiform | 19 | 2.2361 | SEEG |
| Left Inferior Temporal | 19 | 1 | SEEG |
| Left Inferior Temporal | 19 | 2 | SEEG |
| Left Inferior Temporal | 19 | 4.3589 | SEEG |
| Left Inferior Temporal | 19 | 5.1962 | SEEG |
| Left Inferior Temporal | 19 | 7.4833 | SEEG |
| Left Lateral Orbitofrontal | 19 | 1.4142 | SEEG |
| Left Middle Temporal | 19 | 3.1623 | SEEG |
| Left Middle Temporal | 19 | 6.7082 | SEEG |
| Left Pars Orbitalis | 19 | 1 | SEEG |
| Left Caudal Middle Frontal | 26 | 2.2361 | SEEG |
| Left Caudal Middle Frontal | 26 | 3.7417 | SEEG |
| Left Fusiform | 26 | 3.7417 | SEEG |
| Left Fusiform | 26 | 4 | SEEG |
| Left Fusiform | 26 | 9.0554 | SEEG |
| Left Inferior Temporal | 26 | 5.099 | SEEG |
| Left Middle Temporal | 26 | 2.4495 | SEEG |
| Left Middle Temporal | 26 | 3.6056 | SEEG |
| Left Pars Orbitalis | 26 | 0 | SEEG |
| Left Pars Orbitalis | 26 | 1 | SEEG |
| Left Precentral | 26 | 5.4772 | SEEG |
| Left Superior Temporal | 26 | 4 | SEEG |
| Right Caudal Anterior Cingulate | 26 | 1.4142 | SEEG |
| Right Caudal Middle Frontal | 26 | 1 | SEEG |
| Right Caudal Middle Frontal | 26 | 3 | SEEG |
| Right Caudal Middle Frontal | 26 | 3.1623 | SEEG |
| Right Caudal Middle Frontal | 26 | 4.3589 | SEEG |
| Right Fusiform | 26 | 1 | SEEG |
| Right Fusiform | 26 | 3.1623 | SEEG |
| Right Inferior Temporal | 26 | 1.7321 | SEEG |
| Right Inferior Temporal | 26 | 3.7417 | SEEG |
| Right Inferior Temporal | 26 | 4.1231 | SEEG |
| Right Inferior Temporal | 26 | 5.9161 | SEEG |
| Right Insula | 26 | 3 | SEEG |
| Right Insula | 26 | 3.4641 | SEEG |
| Right Precentral | 26 | 2.4495 | SEEG |
| Right Precentral | 26 | 2.8284 | SEEG |
| Right Precentral | 26 | 3.7417 | SEEG |
| Right Precentral | 26 | 7.5498 | SEEG |
| Right Precuneus | 26 | 1.4142 | SEEG |
| Right Precuneus | 26 | 4.5826 | SEEG |
| Right Rostral Middle Frontal | 26 | 0 | SEEG |
| Right Rostral Middle Frontal | 26 | 2.2361 | SEEG |
| Right Superior Frontal | 26 | 1 | SEEG |
| Right Superior Frontal | 26 | 1.4142 | SEEG |
| Right Superior Parietal | 26 | 2.4495 | SEEG |
| Right Superior Parietal | 26 | 3.1623 | SEEG |
| Right Superior Parietal | 26 | 5.3852 | SEEG |
| Left Fusiform | 18 | 1 | SEEG |
| Left Fusiform | 18 | 3.1623 | SEEG |
| Left Middle Temporal | 18 | 2.2361 | SEEG |
| Right Inferior Temporal | 18 | 1.7321 | SEEG |
| Right Middle Temporal | 18 | 1.7321 | SEEG |
| Right Precuneus | 18 | 3.6056 | SEEG |
| Right Superior Frontal | 18 | 1.4142 | SEEG |
| Right Superior Frontal | 18 | 2 | SEEG |
| Right Superior Frontal | 18 | 2.2361 | SEEG |
| Right Superior Frontal | 18 | 4.2426 | SEEG |
| Right Superior Parietal | 18 | 2.2361 | SEEG |
| Right Superior Temporal | 18 | 2.2361 | SEEG |
| Left Caudal Middle Frontal | 8 | 0 | Grid |
| Left Pars Opercularis | 8 | 0 | Grid |
| Left Pars Orbitalis | 8 | 0 | Grid |
| Left Pars Triangularis | 8 | 0 | Grid |
| Left Postcentral | 8 | 0 | Grid |
| Left Precentral | 8 | 0 | Grid |
| Left Rostral Middle Frontal | 8 | 0 | Grid |
| Left Superior Temporal | 8 | 0 | Grid |
| Left Supramarginal | 8 | 0 | Grid |
| Left Transverse Temporal | 8 | 0 | Grid |
